# Inflammatory responses following CRISPR modification of the nuclear localisation sequence in endogenous interleukin-1 alpha

**DOI:** 10.1242/dmm.052705

**Published:** 2026-04-13

**Authors:** Christopher Hoyle, Rodrigo Díaz Pino, Si Min Lai, Jack P. Green, Antony Adamson, Graham Coutts, Catherine B. Lawrence, Mark Travis, David Brough, Gloria Lopez-Castejon

**Affiliations:** ^1^ Division of Neuroscience, School of Biological Sciences, Faculty of Biology, Medicine and Health, University of Manchester, Manchester Academic Health Science Centre, Manchester, M13 9PT, UK; ^2^ Geoffrey Jefferson Brain Research Centre, The Manchester Academic Health Science Centre, Northern Care Alliance NHS Group, University of Manchester, Manchester, M13 9PT, UK; ^3^ The Lydia Becker Institute of Immunology and Inflammation, University of Manchester, Manchester, M13 9PT, UK; ^4^ Division of Infection, Immunity and Respiratory Medicine, School of Biological Sciences, Faculty of Biology, Medicine and Health, University of Manchester, Manchester Academic Health Science Centre, Manchester, M13 9PT, UK; ^5^ Genome Editing Unit, Faculty of Biology, Medicine and Health, University of Manchester, Manchester, M13 9PT, UK

**Keywords:** Interleukin-1 alpha, Nuclear localisation, Inflammation, Cytokine, Influenza

## Abstract

Interleukin (IL)-1α is a pro-inflammatory member of the IL-1 cytokine superfamily and is important for inflammatory responses to infection and injury. Unlike pro-IL-1β, pro-IL-1α is mainly localised to the nucleus upon expression. This is mediated by a nuclear localisation sequence (NLS) responsible for its importin-dependent transport into the nucleus. This nuclear localisation and the presence of histone acetyl transferase (HAT)-binding domains within the pro-domain suggest a role of this cytokine in gene transcription regulation. In addition, nuclear trafficking of pro-IL-1α is proposed to regulate its secretion. To date, studies on the nuclear role of pro-IL-1α have used overexpression systems. Here, we generated a mouse strain in which the endogenous *Il1a* gene was edited using CRISPR to disrupt the NLS, yielding a mutated NLS (mNLS). Using an *in vitro* approach with murine macrophages we found that this NLS mutation did not affect pro-IL-1α RNA expression levels in response to lipopolysaccharide (LPS) but increased its protein expression levels. Moreover, we found that the transcriptional signature induced by LPS was not altered between WT and mNLS macrophages. Release of IL-1α in response to different stimuli such as ionomycin was not negatively impacted by disrupted nuclear localisation, although higher levels of IL-1α release were detected, potentially due to increased levels of pro-IL-1α. Inflammatory responses in an *in vivo* model of peritonitis and an influenza infection model were comparable between WT and mNLS mice. Thus, we have established a mouse model in which pro-IL-1α nuclear localisation is disrupted, although future research is required to reveal the importance of this nuclear localisation for IL-1α function.

## Teaser

**Summary:** A mouse model with disrupted nuclear localization of pro-IL-1α shows no differences in the inflammatory responses compared to WT mice, neither *in vitro* nor *in vivo*. Future research is needed to reveal the importance of this nuclear localisation for IL-1α function.

## INTRODUCTION

Interleukin-1α (IL-1α) is a pro-inflammatory member of the IL-1 cytokine superfamily, and is important for inflammatory responses to infection and injury (Cavalli et al., 2021). IL-1α is produced as a 31 kDa precursor called pro-IL-1α, proteolytic cleavage of which produces a 17 kDa mature protein of increased biological activity that is released and signals via the type 1 IL-1 receptor (IL-1R1) on neighbouring cells (Cavalli et al., 2021). IL-1α is closely related to IL-1β, another proinflammatory member of the IL-1 family, and from which it arose as a gene duplication in mammals (Rivers-Auty et al., 2018). Whilst both IL-1α and IL-1β share a receptor through which they can initiate inflammatory signalling (IL-1R1), some additional functionality is attributed to pro-IL-1α due to the presence of a nuclear localisation sequence (NLS) and histone acetyl transferase (HAT)-binding domains within the pro-domain (Rivers-Auty et al., 2018; Buryskova et al., 2004; Zamostna et al., 2012). In general, the pro-domain of pro-IL-1α and its NLS is well conserved amongst most mammals, although it is lost in a number of species (Rivers-Auty et al., 2018; Wellens et al., 2024). When intact, the NLS on pro-IL-1α is responsible for its importin-dependent transport into the nucleus (Luheshi et al., 2009a; Yamada et al., 2024).

In addition to nuclear actions, we have also hypothesised previously that nuclear trafficking of pro-IL-1α can act to regulate its secretion. Unlike pro-IL-1β, pro-IL-1α is not directly activated by caspase-1 following inflammasome activation. Cleavage depends upon Ca^2+^-activated calpain proteases (Tapia et al., 2019), which become active in response to stimuli that increase intracellular Ca^2+^ concentration, including inflammasome activators like extracellular ATP, although other proteases have also been described (Wiggins et al., 2019; Afonina et al., 2015). Once processed, mature IL-1α is secreted via unconventional protein secretion pathways (Tapia et al., 2019) or is released upon cell death (Luheshi et al., 2009b). We have previously reported that, in COS7 cells transfected to express either pro-IL-1α-GFP or GFP alone, the release of IL-1α is limited by its retention in the nucleus (Luheshi et al., 2009b). Furthermore, in HeLa cells that had been transiently transfected to express wild type (WT) pro-IL-1α or pro-IL-1α in which the NLS was mutated to lose function and, hence, mainly present in the cytosol, more IL-1α is secreted from cells expressing the mutant in response to IL-1α secretion stimuli, such as the Ca^2+^ ionophore ionomycin (Wellens et al., 2024).

IL-1α is important for an effective antiviral host-response, particularly where the virus has host evasion mechanisms. HSV-1 infection inhibits the release of IL-1β via an immune evasion mechanism and IL-1α is essential for a protective immune response (Milora et al., 2014). Orzalli et al. established that, in response to viruses that can evade pattern recognition receptor (PRR)-dependent antiviral gene expression, the IL-1 cytokines IL-1α and IL-1β provide a back-up antiviral system, during which these cytokines induce expression of antiviral genes and limit viral replication (Orzalli et al., 2018). In a follow-up study, Orzalli et al. suggest that, in human keratinocytes, viral disruption of protein synthesis within the host cell activates guard proteins, thereby leading to a gasdermin-E-dependent pyroptosis and IL-1α release (Orzalli et al., 2021). Thus, losing the NLS could benefit an organism fighting viral infection by increasing levels of released IL-1α. This was our rationale for the following study, where we aimed to establish the importance of nuclear trafficking during inflammation and its potential importance for effective antiviral inflammatory responses.

## RESULTS

### Mutation of pro-IL-1α NLS leads to loss of nuclear localisation

**Fig. 1.**
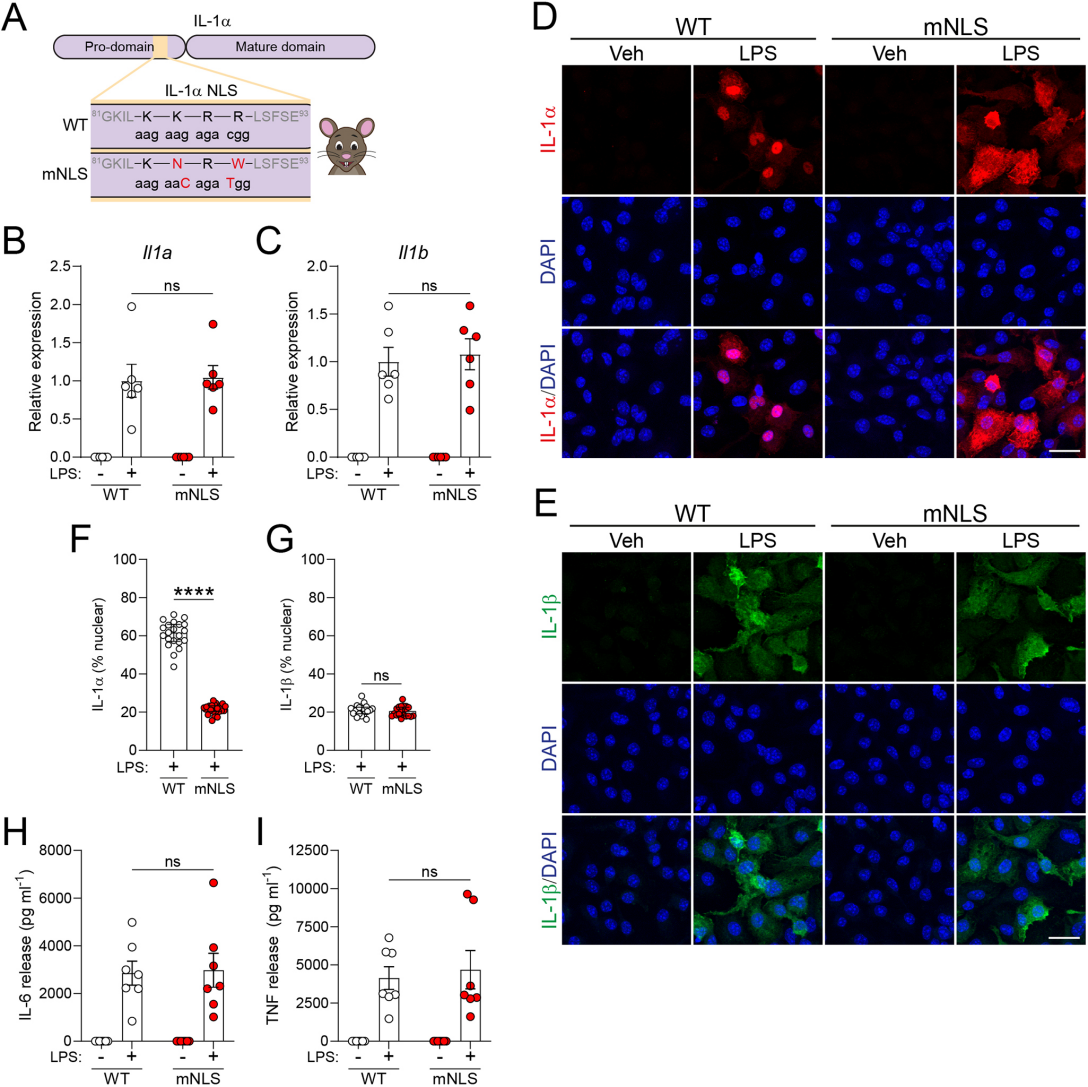
**Pro-IL-1α NLS mutation does not affect expression but reduces nuclear localisation.** (A) Schematic of pro-IL-1α NLS mutation in the mNLS mouse. The NLS motif constituted by amino acids Lys-Lys-Arg-Arg (KKRR) as positions 85-88 was mutated to Lys-Asn-Arg-Trp (KNRW) through two base substitutions. (B-I) WT or mNLS primary BMDMs were primed with vehicle (PBS) or LPS (1 μg/ml, 4 h). qPCR analysis of *Il1α* (B) and *Il1β* (C) mRNA expression from cell lysates (*n*=6), showing similar expression levels between WT and mNLS BMDMs in response to LPS. BMDMs were analysed by confocal microscopy images of immunofluorescence labelling of nuclei (DAPI, blue) and pro-IL-1α (red) (D) or pro-IL-1β (green) (E) using antibody labelling against IL-1α and IL-1β, with maximum intensity projections shown. Pro-IL-1α was localised to the nucleus in the WT BMDMs, which was lost in the mNLS BMDMs. Pro-IL-1β was predominantly cytosolic in both cell types. Nuclear localisation of pro-IL-1α (F) and pro-IL-1β (G) was quantified (*n*=7, with 21 (WT) or 22 (mNLS) fields of view quantified. Scale bars: 20 µm. Release of IL-6 (H) and TNF (I) into the supernatant (*n*=7) following cell treatment, showing similar release from WT and mNLS BMDMs in response to LPS, indicating that TLR4 signalling was unaffected. n numbers indicate biological replicates. Cytokine release was measured using ELISA. Data are presented as mean±s.e.m. (B,C,H,I) or the median±IQR (F,G). Data were analysed using two-tailed unpaired *t*-test (C,F,G,H) or two-tailed Mann–Whitney test (B,I). ****P<0.0001; ns, not significant.

Our previous studies on mutating the NLS to investigate its effect on secretion of IL-1α relied upon ectopic expression systems (Wellens et al., 2024; Luheshi et al., 2009b). Here, we wanted to study the effect of nuclear trafficking on endogenous IL-1α and, to do this, we employed CRISPR to mutate the NLS. In our first attempt, we generated mice with mutations in the NLS by using a CRISPR strategy that resulted in seven base substitutions (Daniels et al., 2017). These substitutions were combinations of the desired mutations and some synonymous, silent mutations intended to prevent re-editing of the allele and make detectable changes for follow-on colony genotyping. However, analysis of these mice showed a loss of *Il1a* gene expression. Further analysis of the gene locus indicated that the combination of substitutions likely impacted the sequence and structure of a long non-coding RNA (lncRNA) that has been found antisense to *Il1a* (now named AS-IL1α), and which has cis-regulatory functions for *Il1a* itself (Chan et al., 2015). To prevent this collateral gene perturbation, we designed a second strategy to minimise the number of base substitutions generated, creating just two base changes in the NLS-encoding residues. We used the RNAsnp web werver (https://rth.dk/resources/rnasnp/) to predict the impact of these changes on the AS-IL1α structure, which indicated no significant structural change. The two base substitutions mutated a critical motif within the NLS of the endogenous pro-IL-1α constituted by amino acids (aa) Lys-Lys-Arg-Arg at positions 85-88 (^85^KKRR^88^) to the non-functional NLS sequence constituted by amino acids (aa) Lys-Asn-Arg-Trp at positions 85-88 (^85^KNRW^88^) that is shared by almost all toothed whales, previously been shown by us to prevent IL-1α nuclear localisation in an ectopic expression system (Wellens et al., 2024) (Fig. 1A, Fig. S1). Our mutant mice are, therefore, called mutant NLS (hereafter, mNLS) mice.

Bone marrow-derived macrophages (BMDMs) were isolated and cultured from WT and mNLS mice, treated with or without lipopolysaccharide (LPS) and subsequently analysed by qPCR. We confirmed that both IL-1α and IL-1β were expressed, and that expression levels were similar between WT and mNLS BMDMs (Fig. 1B,C). We also analysed the levels of *Il1a* and *Il1b* mRNA expression after different durations of LPS treatment. There were no clear differences in *Il1a* or *Il1b* mRNA levels between WT and mNLS, suggesting comparable mRNA stability at these time points (Fig. S2). A similar experiment was conducted but the cells were fixed and immunocytochemistry was used to analyse pro-IL-1α and pro-IL-1β expression and cellular localisation. In WT mice, pro-IL-1α was largely located in the nucleus, as expected, while pro-IL-1β was diffusely present throughout the cell (Fig. 1D-G). Strikingly, in mNLS mice the pro-IL-1α was no longer concentrated in the nucleus but was diffusely distributed throughout the cell, similar to pro-IL-1β (Fig. 1D-G). LPS-induced release of IL-6 and TNF was similar between WT and mNLS BMDMs, indicating that TLR4 signalling was unaffected (Fig. 1H,I). We also examined pro-IL-1α subcellular localisation in peritoneal macrophages isolated from these mice that had subsequently been primed with LPS, and detected nuclear pro-IL-1α in WT mice and cytosolic pro-IL-1α in mNLS mice (Fig. S3). These data show that our mNLS mice expressed normal levels of pro-IL-1α, and that the mutations of the NLS had disrupted the nuclear localisation of pro-IL-1α.

**Fig. 2.**
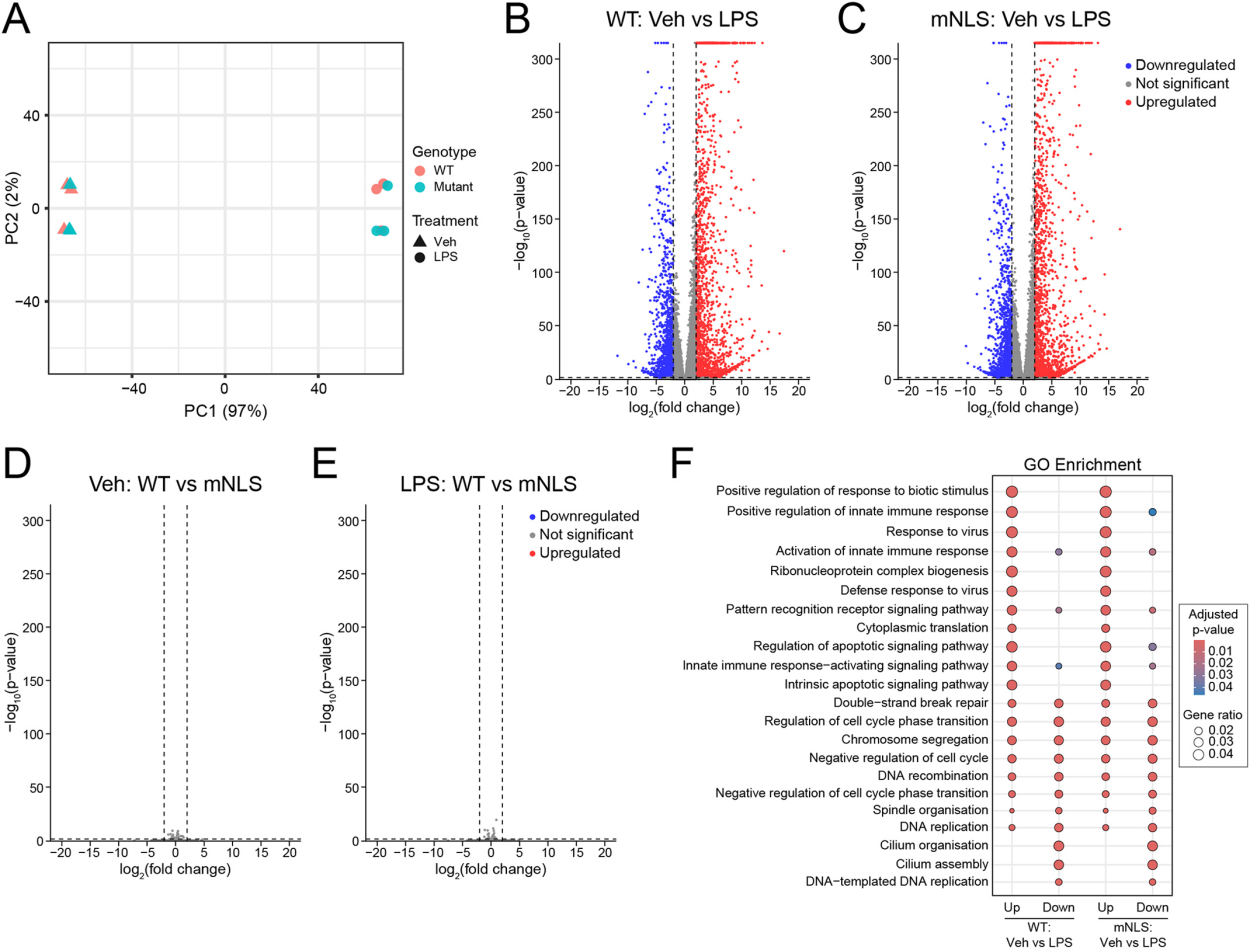
**mNLS IL-1α does not affect LPS-induced gene expression in BMDMs.** WT or mNLS BMDMs were primed with vehicle (PBS) or LPS (1 µg/ml, 4 h) (*n*=3), followed by bulk RNA-sequencing (RNA-Seq) analysis. (A) Principal component analysis (PCA) of RNA-Seq data showing clustering by genotype and treatment. A clear treatment effect was observed, but no genotype effect. (B) Volcano plot of differentially expressed genes in WT BMDMs [PBS (Veh) versus LPS treatment]. (C) Volcano plot of differentially expressed genes in mNLS BMDMs (Veh versus LPS). (D) Volcano plot of differentially expressed genes between WT and mNLS BMDMs after vehicle treatment. (E) Volcano plot of differentially expressed genes between WT and mNLS BMDMs after LPS treatment. LPS treatment induced a large transcriptional response compared with vehicle treatment in WT and mNLS BMDMs, although there were no differences in this response between WT and mNLS BMDMs. (F) Gene ontology (GO) enrichment analysis of biological processes for differentially expressed genes in WT (Veh versus LPS, left) and mNLS (Veh versus LPS, right) BMDMs. *n* numbers indicate biological replicates. Data were analysed as described under Materials and Methods.

As nuclear IL-1α is proposed to influence transcriptional regulation (Werman et al., 2004), we next performed bulk RNA sequencing in BMDMs treated with PBS or LPS. We observed that LPS induced a large transcriptional response in WT and mNLS BMDMs (Fig. 2A-C); however, there were no differences in this response between WT and mNLS cells (Fig. 2D-F).

### Loss of nuclear localisation does not impair pro-IL-1α processing and release

**Fig. 3.**
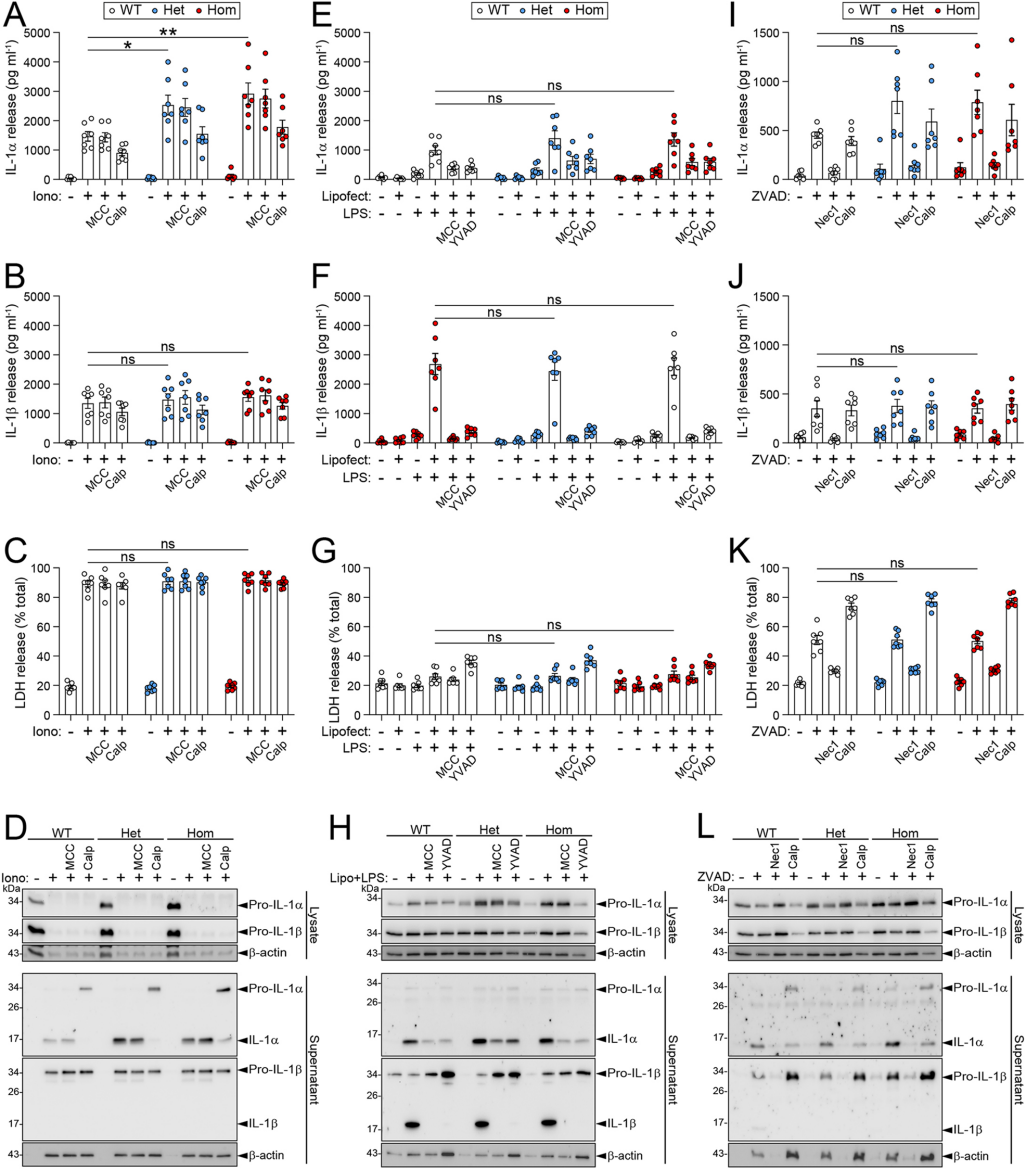
**Pro-IL-1α NLS mutation does not negatively affect processing and release of IL-1α.** (A-D) WT or mNLS BMDMs were primed with LPS (1 µg/ml, 4 h), followed by ionomycin treatment (10 µM, 1 h) in the presence or absence of MCC950 (10 µM; MCC) or calpeptin (40 µM; Calp) (*n*=7). Supernatants were assessed for IL-1α release (A), IL-1β release (B) and LDH release (C). Cell lysates and supernatants were assessed for IL-1α and IL-1β expression and processing by western blotting (D). mNLS BMDMs exhibited robust IL-1α release which tended to be higher than WT BMDMs, while levels of released IL-1β and LDH were similar between WT and mNLS BMDMs. Levels of pro-IL-1α tended to be higher in the mNLS BMDMs in response to LPS priming. (E-H) WT or mNLS BMDMs were primed with Pam3CSK4 (100 ng/ml, 4 h), before transfection of LPS (2 µg/ml, 18 h) in the presence or absence of MCC950 (10 μM) or YVAD (100 μM) (*n*=7). Supernatants were assessed for IL-1α release (E), IL-1β release (F) and LDH release (G). Cell lysates and supernatants were assessed for IL-1α and IL-1β expression, and processing by western blotting (H). Comparable IL-1α and IL-1β processing and release was observed between WT and mNLS cells. (I-L) WT or mNLS BMDMs were primed with LPS (1 µg/ml, 4 h), followed by ZVAD treatment (50 µM, 5 h) in the presence or absence of necrostatin-1 (50 μM; Nec1) or calpeptin (40 μM) (*n*=7). Supernatants were assessed for release of IL-1α (I), IL-1β (J) and LDH (K). Cell lysates and supernatants were assessed for IL-1α and IL-1β expression and processing by western blotting (L). IL-1α release was modestly increased from mNLS BMDMs compared to WT BMDMs, with IL-1β release remaining the same between the cells. *n* numbers indicate biological replicates. Cytokine release was measured using ELISA. Data are presented as mean±s.e.m. Data were analysed using one-way ANOVA followed by Dunnett's post hoc test (A-C,E,F,I-K) or Kruskal–Wallis test followed by Dunn's post hoc test (G). *P<0.05; **P<0.01; ns, not significant.

Having shown that mNLS IL-1α did not affect LPS-induced gene expression, we set out to determine whether disrupted nuclear localisation of pro-IL-1α affected its processing and release. BMDMs were treated with LPS to induce IL-1α expression, and then treated with ionomycin to induce calpain-dependent processing and release of IL-1α. mNLS BMDMs exhibited robust IL-1α release in response to ionomycin; this tended to be higher than in WT BMDMs, while levels of released IL-1β and LDH release were unaffected between WT and mNLS cells (Fig. 3A-C). As expected, the IL-1β released was almost exclusively pro-IL-1β in response to cell death (Fig. 3D, Fig. S4), indicating a lack of NLRP3 inflammasome activation in response to ionomycin. Levels of pro-IL-1α protein in the cell lysates were higher in the mNLS BMDMs in response to LPS priming, potentially contributing to the observed enhanced levels of IL-1α cleavage and release (Fig. 3D, Fig. S4). No differences in IL-1α or IL-1β release were observed between WT and mNLS BMDMs when different concentrations of ionomycin were used (Fig. S4G-I).

IL-1α has also been reported to be released from macrophages in response to canonical NLRP3 inflammasome activation (Gross et al., 2012), non-canonical inflammasome activation (Wiggins et al., 2019) and in response to necroptosis (England et al., 2014). Canonical NLRP3 inflammasome activation triggers Ca^2+^ entry through gasdermin D membrane pores, which activates calpain-dependent IL-1α processing and release (Tsuchiya et al., 2021). In response to treatment with LPS followed by treatment with the canonical NLRP3 activators nigericin and ATP, IL-1α was processed and released from mNLS BMDMs, and this release was modestly increased compared to that from WT BMDMs (Fig. S5A,D). This release was inhibited by the NLRP3 inhibitor MCC950, suggesting it is occurring downstream of NLRP3 inflammasome activation (Fig. S5A,D). IL-1β release induced by nigericin or ATP was consistent between WT and mNLS BMDMs (Fig. S5B,E). No differences in IL-1α or IL-1β release were observed between WT and mNLS BMDMs when different concentrations of ATP were used (Fig. S5G-I). Activation of the non-canonical inflammasome, stimulated by transfection with LPS which could be blocked by MCC950 as well as caspase-1 inhibitor YVAD, caused comparable IL-1α and IL-1β processing, and release from WT and mNLS BMDMs (Fig. 3E-H, Fig. S6A-F). The induction of necroptosis in response to LPS and ZVAD treatment also induced IL-1α release from mNLS BMDMs, which was modestly increased compared to that from WT BMDMs and with, again, levels of IL-1β release remaining the same between the cells (Fig. 3I-L, Fig. S6G-L). Cell death, as measured by LDH release, was the same between WT and mNLS BMDMs across all experiments and stimuli. Together, these data suggest that loss of pro-IL-1α nuclear localisation does not negatively impact its processing and release. Furthermore, these data suggest that nuclear localisation may limit IL-1α release – consistent with previously published data utilising ectopic overexpression systems – and that, in species lacking the NLS, one may predict greater levels of IL-1α release during inflammation.

### mNLS mutation does not affect IL-1α release in an *in vivo* model of peritoneal inflammation

**Fig. 4.**
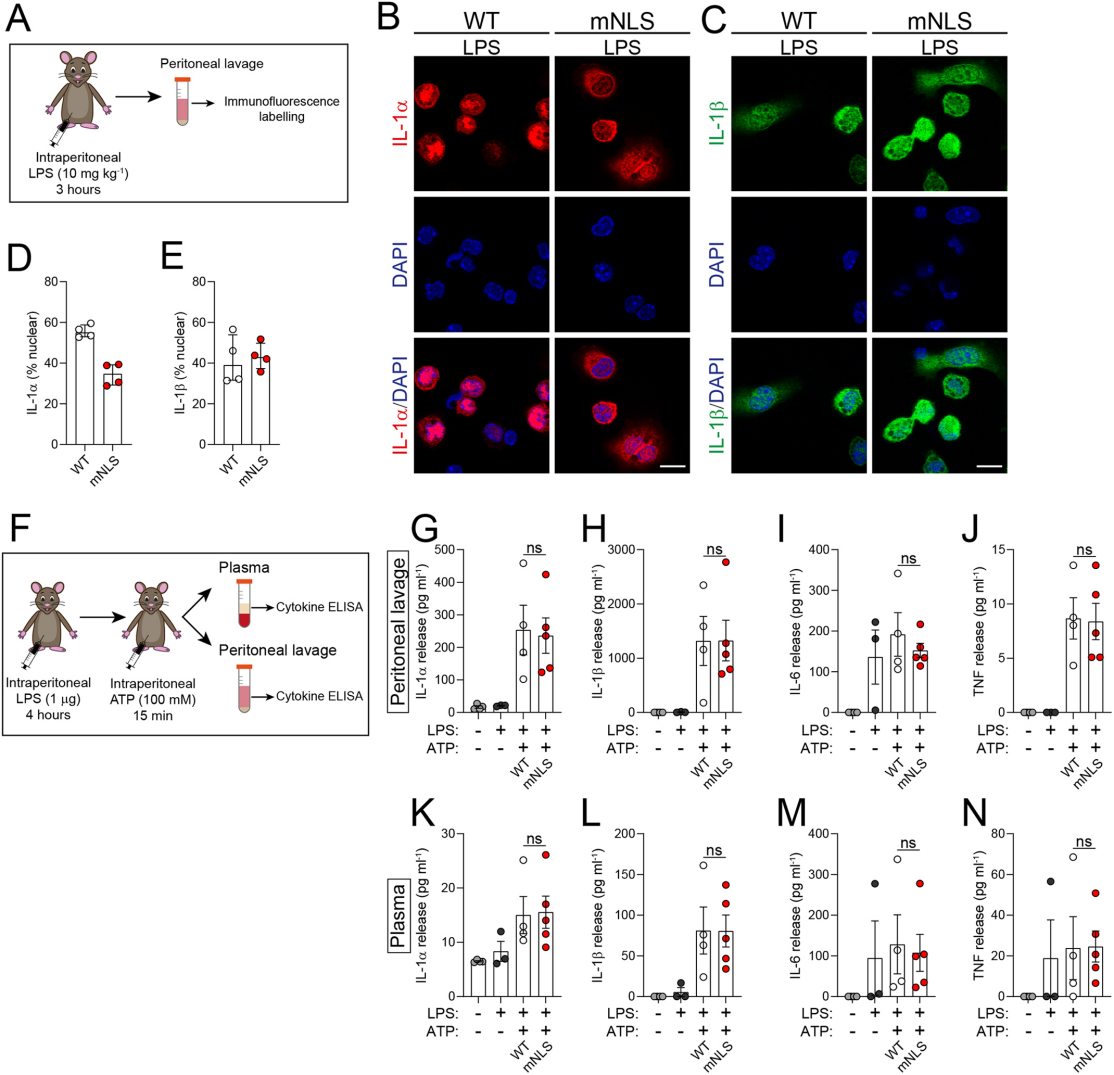
**mNLS mutation did not affect IL-1α release in an *in vivo* model of peritoneal inflammation.** (A) Schematic of LPS injection model. (B-E) WT (*n*=2) and mNLS (*n*=2) mice were injected with LPS (10 mg/kg, intraperitoneal) for 3 h, after which peritoneal lavage was collected. Peritoneal lavage cells were isolated, adhered to coverslips, fixed and analysed by confocal microscopy of immunofluorescence labelling of nuclei (DAPI, blue) and pro-IL-1α (red) (B) or pro-IL-1β (green) (C) using antibody labelling against IL-1α and IL-1β, with a single z-plane shown. Nuclear IL-1α signal was observed in peritoneal cells from WT mice, but this was lost in peritoneal cells from mNLS mice. IL-1β signal was predominantly cytosolic in peritoneal cells from WT and mNLS mice. Nuclear localisation of pro-IL-1α (D) and pro-IL-1β (E) was quantified. Quantified were four fields of view each for WT and mNLS. Scale bars: 10 µm. (F-N) WT (*n*=4) and mNLS (*n*=5) mice were injected with LPS (1 µg, intraperitoneal) for 4 h, followed by ATP (100 mM, intraperitoneal under anaesthesia), after which plasma and peritoneal lavage were collected. Heterozygous control mice were left untreated (*n*=4) or injected with LPS (1 µg, intraperitoneal) for 4 h, followed by PBS (*n*=3). (F) Schematic of LPS+ATP injection model. (G-N) Cytokine release was measured in peritoneal lavage (G-J) and plasma (K-N). *n* numbers indicate number of individual mice. LPS and ATP treatment increased the release of IL-1α, IL-1β, IL-6 and TNF from WT and mNLS mice, with no difference observed between the strains. Cytokine release was measured using ELISA. Data are presented as mean±s.e.m. (G-N) or median±IQR (D,E). Data were analysed using two-tailed unpaired *t*-test (G,H,I,J,L,N) or two-tailed Mann–Whitney test (K,M). ns, not significant.

As our *in vitro* data pointed to enhanced IL-1α expression and release levels after LPS treatment, we next performed an LPS intraperitoneal injection model (Baldwin et al., 2017) (Fig. 4A). We tested whether, consistent with our *in vitro* experiments, immune cells isolated from LPS-injected WT or mNLS mice still exhibited the nuclear or cytosolic localisation of pro-IL-1α. We observed that peritoneal immune cells, obtained from the peritoneal cavity of WT mice after LPS injection, presented a predominantly nuclear localisation of IL-1α, while immune cells isolated from mNLS mice presented a mainly cytosolic IL-1α localisation (Fig. 4B,D). IL-1β exhibited a cytosolic localisation in cells from both WT and mNLS mice (Fig. 4C,E).

Next, we used an *in vivo* model of NLRP3-dependent inflammation where mice were administered with LPS followed by treatment with the NLRP3-activating stimulus ATP to induce NLRP3-dependent IL-1β release (Griffiths et al., 1995; Solle et al., 2001; Perregaux et al., 2001), and also IL-1α release, as previously reported by us when using this model (Daniels et al., 2016). Thus, to determine whether mNLS mice secrete more IL-1α in response to inflammatory stimuli compared to WT mice *in vivo*, we intraperitoneally injected mice with LPS followed by injection with ATP, and assayed the peritoneal lavage fluid and the plasma for IL-1α and other cytokines (Fig. 4F). In the peritoneal lavage fluid, LPS and ATP increased the release of IL-1α and IL-1β from WT and mNLS mice, and there were no differences between the strains (Fig. 4G,H). Likewise, induced levels of IL-6 and TNFα were the same across both strains (Fig. 4I,J), and this was also reflected in the plasma (Fig. 4K-N). Thus, under these conditions the release of IL-1α was not influenced by the NLS.

### Immune response to influenza infection *in vivo* shows no altered response in mNLS compared to WT mice

**Fig. 5.**
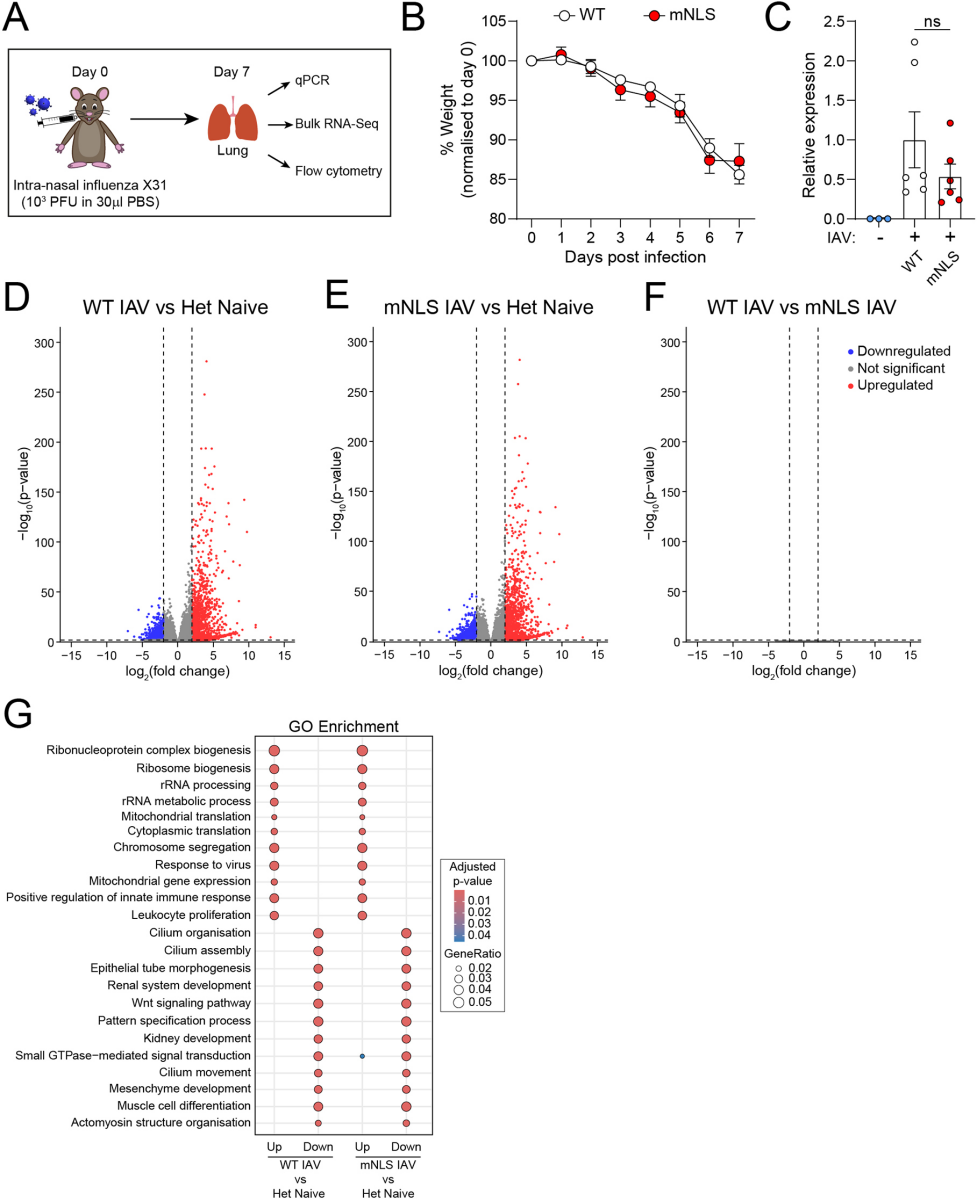
**Pro-IL-1α mutation does not modulate the immune response in an influenza infection model.** (A) On day 0, WT (*n*=6) or mNLS (*n*=6) mice were infected intranasally with live influenza strain X31 (10^3^ PFU in 30 µl of PBS), while heterozygous (Het) mice (*n*=3) were left uninfected (naïve) as a control. (B) Weight loss was monitored between days 0 and 7. Influenza infection caused gradual weight loss, with no difference between WT and mNLS mice. (C) On day 7, the mice were culled and viral load in the lungs was measured via qPCR, with no significant difference detected between WT and mNLS mice. (D-F) Bulk RNA-sequencing (RNA-Seq) analysis was performed on lung samples to assess gene expression, with differences between the groups expressed using volcano plots. The groups compared were infected WT versus uninfected heterozygous (D), infected mNLS versus uninfected heterozygous (E), and infected WT versus infected mNLS (F). (G) Gene ontology (GO) enrichment analysis of biological processes for differentially expressed genes in WT influenza A virus (IAV) versus Het naïve (left) and mNLS IAV versus Het naïve (right). The transcriptional profile observed in the lung was strongly antiviral, with no differences between WT and mNLS mice. *n* numbers indicate number of individual mice. Data are presented as mean±s.e.m. Data were analysed using two-way ANOVA (B) or two-tailed unpaired *t*-test (C). ns, not significant.

We hypothesised that loss of IL-1α nuclear localisation can influence outcomes of viral infection. Here, we used the H3N2 (X31) influenza virus for infection, as IL1a plays a key role in initiating the immune response to control this pathogen. For this experiment, WT or mNLS mice were infected for seven days, because at this timepoint viral load is increased and T cell numbers are reduced in IL-1R1 KO compared to WT mice (Schmitz et al., 2005) (Fig. 5A). We also included uninfected heterozygote control mice. Influenza infection caused a gradual loss of weight over seven days, indicating progression of infection; however, infected WT and mNLS mice lost an equivalent amount of weight over this period (Fig. 5B). When viral load was measured in the lung at day 7, no statistically significant differences were detected between WT and mNLS mice (Fig. 5C). As expected, different immune cell subsets were recruited to the lung at seven days post infection (Figs S7 and S8); however, as with previous markers no significant differences were found between WT and mNLS infected mice.

As pro-IL-1α could have a transcriptional role via regulation of HAT function (Buryskova et al., 2004), and because enhanced secretion of IL-1α could impact the transcriptional profile via IL-1R1, we conducted RNA-sequencing (RNA-Seq) analyses of lung tissue from uninfected heterozygote control mice, and X31-infected WT and mNLS mice. The transcriptional profile observed in the lungs of infected WT and mNLS mice was, as expected, strongly antiviral, and there were no differences between the strains (Fig. 5D-G). These data suggest that loss of IL-1α nuclear localisation does not provide an immune advantage in the protection from influenza infection, at least at the timepoint tested here.

## DISCUSSION

IL-1α, unlike IL-1β, localises to the cell nucleus, driven by the presence of an NLS in its pro-domain. Here, we generated a mouse strain that contains two mutations in this NLS (i.e. mNLS mice), rendering its nuclear transport inactive. We validated this loss of nuclear localisation both *in vitro* and *ex vivo* and confirmed that upon LPS treatment, mNLS IL-1α was present in the cytosol while IL-1β localisation was not affected. Similarly, our data showed that IL-1α levels were not altered by the mNLS at the transcriptional level but were increased at the protein level, suggesting that NLS mutations affect protein stability.

Nuclear IL-1α has been proposed to be a transcriptional regulator that can bind to HATs (Buryskova et al., 2004; Wellens et al., 2024) and is able to regulate inflammatory gene expression, including IL-6 (Werman et al., 2004). However, in our experimental models no changes in IL-6, TNF-α or any other transcriptional changes were observed after LPS priming in mNLS mice. One possibility is that any transcriptional changes mediated by pro-IL-1α in response to LPS may occur at later time points, as pro-IL-1α itself will first need to accumulate in the cell.

IL-1α plays an important role in response to pathogenic infections, including viruses. IL-1α is a key mediator of the innate immune response triggered by dsDNA in adenoviruses, and also in the response to influenza A virus (IAV) infection, by triggering induction of IL-1β and the consequent immune response to infection (Momota et al., 2020). We speculated that loss of the IL-1α NLS, as seen in other species, such as toothed whales, may modify the immune response to infection (Rivers-Auty et al., 2018; Wellens et al., 2024). Toothed whales have lost functional *Mx1* and *Mx2* genes from their genome (Braun et al., 2015), which encode MX1 and MX2 respectively, two dynamin-like GTPase proteins that are important for antiviral responses (Zav'yalov et al., 2019). Braun et al. hypothesised that the evolutionary pressure driving the loss of Mx proteins from the toothed whale arose from a virus that exploited Mx function, such as an ancestor of the herpes simplex virus 1 (Braun et al., 2015). They proposed that, in order to compensate for the loss of Mx proteins, other mutations may have occurred elsewhere in the toothed whale genome. Based on this, we speculated that such a mutation is the mutation of the NLS found in pro-IL-1α. However, in our X31 influenza-infection model, we did not detect any significant differences in weight loss or in the transcriptional profile in the lung between WT and mNLS mice. Infected mNLS mice tended to express higher levels of the T cell activation co-stimulatory markers CD80 and CD86 in alveolar macrophages compared to infected WT mice (Fig. S5), and viral load showed a trend to be reduced in mNLS; however, these findings were statistically insignificant. It is possible that effects of IL-1α in the mNLS mice are more important at different time points post infection. For instance, a reduction in IL-1β secretion levels in IL-1α KO mice was detected at 3 days post infection (Momota et al., 2020). At this time point, cellular damage is higher than at the time point used in our study (7 days post infection). Similarly, it is possible that the nuclear role of IL-1α leads to epigenetic changes that do not impact the outcome of an initial infection but affect the response to a subsequent infection or insult.

Although the mNLS strongly reduced the nuclear localisation of pro-IL-1α by preventing its active trafficking into the nucleus, the mutation did not prevent its passive diffusion into the nucleus. Thus, low levels of mNLS pro-IL-1α were still detected in the nucleus. These low levels of nuclear pro-IL-1α in the mNLS mice may have been sufficient to retain any nuclear activity of pro-IL-1α, possibly explaining the lack of a phenotype observed in many assays. Furthermore, other manipulations of the NLS, such as replacing it with a nuclear export signal, may provide an alternative strategy to investigate the importance of nuclear pro-IL-1α.

One proposed explanation of why pro-IL-1α is localised to the nucleus is that it might prevent excessive release upon necrotic cell death, limiting the impact of the alarmin in the microenvironment (Luheshi et al., 2009b). When we assessed release of IL-1α in response to both inflammasome-dependent and -independent activation stimuli, we observed a trend of increased release of IL-1α from mNLS BMDMs compared to WT BMDMs when saturating concentrations of ionomycin and ATP were used. However, when using lower concentrations of these activating stimuli, these trends in IL-1α release were not detected. However, although no differences in transcriptional IL-1α levels were observed upon LPS priming, higher levels of pro-IL-1α protein were observed in mNLS BMDMs. This could explain the above described increased release of IL-1α from mNLS BMDMs.

Pro-IL-1α can be modified by different post-translational modifications including phosphorylation, ubiquitylation and acetylation (Beuscher et al., 1988; Ainscough et al., 2014; Cohen et al., 2015). Ubiquitylation of pro-IL-1α contributes to protein stability; however, the sites responsible for this modification have not been identified (Ainscough et al., 2014). Hence, it is possible that the K86N mutation within the mNLS motif ^85^KNRW^88^ resulted in decreased ubiquitylation of pro-IL-1α, impacting proteasomal degradation and, hence, affecting IL-1α stability, therefore, resulting in higher protein levels of pro-IL-1α in mNLS BMDMs. Acetylation has been described at K82 within the NLS and proposed to contribute to the nuclear localisation of the protein, since the mutation K82R (acetylation-null version) prevents its nuclear localisation and results in higher secretion levels (Cohen et al., 2015). Interestingly, acetylation can also contribute to protein stability and HATs, such as p300/CBP, can mediate the assembly of polyubiquitin chains (Sadoul et al., 2008). HAT binding to pro-IL-1α could, therefore, also be an important regulator for protein stability in addition to histone modification and gene transcription.

Our work mainly focused on the role of nuclear IL-1α in macrophages, as these cells are important producers of these cytokines. However, pro-IL-1α also plays important roles in non-immune cells, such as keratinocytes, where secretion of IL-1α leads to an IFN-driven signature on skin fibroblasts during viral infection with PRR-evasive viruses, such as VSV (Orzalli et al., 2018). Hence, other inflammatory models that involve non-immune cells and IL-1α should be performed.

Although IL-1β does not localise to the nucleus like pro-IL-1α, other similar cytokines, like IL-33 or IL-37, do (Dinarello, 2018). IL-33 is highly expressed in endothelial and epithelial cells, where it plays important roles during inflammation via ST2 receptors. Similar to IL-1α, IL-33 binds histone and chromatin when it accumulates in the nucleus. However, although IL-33 has initially been proposed to have a transcriptional role, evidence is still limited and the nuclear role for IL-33 is still unclear (Cayrol and Girard, 2018). Whether the nuclear functions of these inflammatory cytokines are conserved still needs to be determined.

Overall, we have generated a new tool to investigate the nuclear role of IL-1α *in vitro* and *in vivo*. Although current experimental models tested here showed no apparent effect on the immune response with strongly reduced levels of nuclear IL-1α, it is possible that nuclear localisation plays a more relevant role at earlier points during infection or in other inflammatory processes during which more subtle roles of IL-1α are required, such as senescence or skin pathologies.

## MATERIALS AND METHODS

### Reagents

Goat anti-mouse IL-1α (AF-400-NA), goat anti-mouse IL-1β (AF-401-NA), mouse IL-1α ELISA (DY400), mouse IL-1β ELISA (DY401), mouse IL-6 ELISA (DY406) and mouse TNF ELISA (DY410) were from R&D Systems. LPS (*in vitro*: L2654; *in vivo*: L4516), ATP (A2383), nigericin (N7143), MCC950 (PZ0280), ZVAD (627610), necrostatin-1 (N9037), bovine serum albumin (A9647), ethylenedinitrilotetraacetic acid disodium salt (EDTA, 03690), deoxyribonuclease I (D4513), Red Blood Cell Lysing Buffer Hybri-Max (R7757), formalin solution, neutral buffered, 10% (HT501128) and anti-β-actin-peroxidase (A3854) were from Sigma-Aldrich. Lipofectamine 3000, Alexa Fluor™ 594 donkey anti-goat IgG (A-11058), DAPI (D1306), ProLong Gold antifade mounting reagent (P36934), eBioscience™ Foxp3 / Transcription Factor Fixation/Permeabilization Concentrate and Diluent (00-5521-00), PureLink RNA mini kit (12183018A) and PureLink DNase set (12185010) were from Invitrogen. YVAD (4018838) was from Cambridge Bioscience. Superscript III Reverse Transcriptase (18080044), Power SYBR™ Green PCR Master Mix (4368706), CountBright™ Absolute Counting Beads (C36950), RNA*later* (AM7020) and TaqMan Gene Expression Assay (FAM) for Hprt1 primers (4331182, Mm03024075_m1) were from Thermo Fisher Scientific. Pam3CSK4 (tlrl-pms) and imiquimod (tlrl-imq) were from InvivoGen. Cytiva Amersham ECL Prime Western Blotting Detection Reagent (RPN2236) was from GE Healthcare. Protease inhibitor cocktail (539131), collagenase D (11088858001), DNase I (11284932001) and formaldehyde solution (252549) were from Merck. CytoTox 96 nonradioactive cytotoxicity assay (G1780) was from Promega. Rabbit anti-goat IgG (P044901-2) secondary antibody was from Agilent. Ionomycin Ca^2+^ salt (CAY11932) was from Cayman Chemical. Collagenase type I (17100017) was from Gibco. RNeasy mini kit (74106) was from Qiagen. High-Capacity RNA-to-cDNA™ Kit (4387406) was from Applied Biosystems. BD Horizon™ Brilliant Stain Buffer Plus (566385), BD Pharmingen™ Purified rat anti-mouse CD16/CD32 (553142, 2.4G2), BD Horizon™ PE-CF594 hamster anti-mouse γδ T-Cell Receptor (563532, E50-2440), BD Horizon™ PE-CF594 rat anti-mouse Siglec-F (562757, E50-2440), BD Horizon™ BUV395 hamster anti-mouse TCR β chain (569248, H57-597), BD Horizon™ BUV496 rat anti-mouse CD4 (612952, GK1.5), BD Horizon™ BUV737 rat anti-CD11b (612800, M1/70) and BD Horizon™ BUV805 rat anti-mouse CD45 (568336, 30-F11) were from BD Biosciences. Zombie UV™ Fixable Viability Kit (423108), Alexa Fluor^®^ 488 anti-mouse CD80 (104716, 16-10A1), PerCP/Cyanine5.5 anti-mouse Ly-6C (128012, HK1.4), APC anti-mouse CD19 (115512, 6D5), Alexa Fluor^®^ 700 anti-mouse Ly-6G (127622, 1A8), APC/Cyanine7 anti-mouse NK-1.1 (108724, PK136), Brilliant Violet 421™ anti-mouse CD64 (FcγRI) (164407, W18349C), Brilliant Violet 510™ anti-mouse/rat XCR1 (148218, ZET), Brilliant Violet 605™ anti-mouse CD86 (105037, GL-1), Brilliant Violet 650™ anti-mouse I-A/I-E (107641, M5/114.15.2), Brilliant Violet 711™ anti-mouse CD24 (101851, M1/69), Brilliant Violet 785™ anti-mouse CD11c (117336, N418), PE anti-mouse CD8a (100708, 53-6.7), PE/Cyanine7 anti-mouse F4/80 (123114, BM8) were from BioLegend.

### mNLS mice generation

Male and female WT and mNLS C57BL/6 mice aged between 3 and 6-months were maintained under standard laboratory conditions: ambient temperatures of 21°C (±2°C), humidity of 40–50%, 12 h light cycle, *ad libitum* access to food and water. All procedures were performed with the observers unaware of the genotype, and were carried out in accordance with the United Kingdom Animals (Scientific Procedures) Act 1986 and approved by the Home Office and the local Animal Ethical Review Group, University of Manchester.

The CRISPR targeting strategy is summarised in Fig. 1A-C. Single guide RNA (sgRNA) 5′-GGAAGATTCTGAAGAAGAGA-3′ that targets the NLS encoding residues of mIL1a was purchased as a chemically synthesised Alt-R oligo (IDT, Coralville, IA, USA). An Alt-R single-stranded oligodeoxynucleotide (ssODN) repair template (5′-TTCAAGGAGAGCCGGGTGACAGTATCAGCAACGTCAAGCAACGGGAAGATTCTGAAGAA**C**AGA**T**GGCTGAGTTTCAGTGAGACCTTCACTGAAGATGACCTGCAGTCCATAACCCATGAT-3′) comprising 60nt homology each side of the NLS sequences was designed to direct two base substitutions (indicated in bold) to mutate the motif KKRR to KNRW. Both sgRNA and ssODN were resuspended in sterile, RNase free injection buffer (Tris-HCl 1 mM pH 7.5, EDTA 0.1 mM) to a concentration of 100 ng/ml. 1 mg of sgRNA guide was mixed with 1 mg Cas9 recombinant protein (NEB) and incubated at RT for 10 mins, before ssODN was added to the injection mix [final concentrations 40 ng/ml; 40 ng/ml respectively, ssODN HDR template (50 mg/ml)]. Cryopreserved 1-cell C57BL/6J embryos from IVF were thawed and cultured for 4 h, before they were pronuclear microinjected with the above mix using standard protocols. Zygotes were cultured overnight, and the resulting two-cell embryos surgically implanted into the oviduct of day 0.5 post-coitum pseudopregnant mice. After birth and weaning, genomic DNA was extracted from ear punches by using the Sigma REDExtract-N-Amp Tissue PCR kit (cat. no.: XNAT-10RXN). DNA was then used to genotype pups by amplifying across the NLS sequence with primers DB27 Geno F1 5′-tcaggttccacttttcctcct-3′ and DB27 Geno R1 5′-TCTTTCCACTCCATTCTACCACT-3′. Amplicons were Sanger sequenced to identify mice harbouring the mutated allele, to confirm the founder mouse and establish a colony. No health or developmental issues were associated with the mNLS mutation, and fertility was normal. mNLS mice were born at Mendelian frequencies (Fig. S1D).

### *In vivo* peritoneal inflammation models

For the LPS-induced peritoneal inflammation model, WT or mNLS littermate mice were administered with LPS (10 mg/kg, from *Escherichia coli* 0127:B8) intraperitoneally (Baldwin et al., 2017). Three hours after injection, mice were anesthetised with isoflurane, and the peritoneal cavity was lavaged with 3 ml of RPMI medium. Peritoneal lavage was centrifuged at 1500 ***g*** for 5 min at 4°C, and the cell pellet was resuspended in DMEM and seeded onto coverslips, left to adhere for 1.5 h, and then fixed and assessed for IL-1α and IL-1β subcellular location by immunofluorescence labelling. One WT mouse and one mNLS mouse were excluded from analysis due a failed intraperitoneal injection, determined by minimal IL-6 detection in the plasma.

For the LPS and ATP-induced peritoneal inflammation model, WT or mNLS littermate mice were administered with LPS (1 μg, from *Escherichia coli* 0127:B8) intraperitoneally (Daniels et al., 2016). Four hours after injection, mice were anesthetised with isoflurane, and injected with ATP (100 mM pH 7.4) for 15 min. Heterozygous control mice were left untreated, or injected with LPS (1 µg) for 4 h followed by PBS for 15 min. Peritoneal lavage was collected as above, and blood was collected via cardiac puncture. Peritoneal lavage was centrifuged at 1500 ***g*** for 5 min at 4°C and the supernatant was analysed for cytokine content. Blood was centrifuged at 1500 ***g*** for 15 min at 4°C, and the supernatant was subsequently centrifuged at 18,000 ***g*** for 3 min at 4°C. The resulting supernatant (plasma) was assessed for cytokine content. One WT mouse was excluded from analysis due a failed intraperitoneal injection, determined by no or minimal IL-1β or IL-6 detection in the plasma or peritoneal lavage.

### *In vivo* influenza infection model

WT or mNLS littermate mice were anaesthetized by 2.5% inhalation isoflurane and intranasally infected with 10^3^ PFU of the influenza A virus (IAV) strain X31 (H3N2), in a volume of 30 µl PBS, on day 0. Mild to moderate weight loss was expected from IAV X31 infection, and daily weights were monitored until day 7 post infection. Infected WT and mNLS mice, and heterozygous control mice, were culled on day 7 post IAV X31 infection. Lung draining (cervical and mediastinal) lymph nodes were first collected, and after PBS perfusion with 10 ml of 1X PBS 1 mM EDTA, three lobes of lung and spleen were collected. The lung and spleen were weighed, minced, and digested with either collagenase type I (10 mg/ml) and DNase I (50 µg/ml) for 40 min (lungs), or with collagenase type D (1 mg/µl) and DNase I (50 µg/ml) for 30 min (spleens), at 37°C in a shaking incubator. The lung draining lymph nodes were counted and sized. The digested lung and spleen tissue, and lung draining lymph nodes, were passed through a 70-µm filter, washed with 25 ml of cold PBS 5 mM EDTA solution, and centrifuged at 500 ***g*** for 5 min. Pelleted cells were resuspended in 1 ml of RBC lysis buffer and washed with 10 ml of PBS. The RBC-lysed single cell suspension was resuspended in complete RPMI media with 10% FCS before flow cytometry staining. No mice were excluded from analysis.

### Flow cytometry

Single cell suspensions (1/5th of lung) were transferred to 5 ml round-bottom polystyrene (FACS) tubes, washed with 3 ml 1× PBS, and resuspended in 300 µl of Zombie UV Live/Dead stain. After 15 min, cells were washed with 3 ml 1× PBS and resuspended in 50 µl of anti-CD16/32 (Fc block) in FACS buffer (2% FCS in PBS 2 mM EDTA). After 10 min, without washing, 10 µl of antibody staining mastermix prepared in BD Brilliant Stain Buffer Plus was added to samples and incubated for 30 min at 4°C in the dark (Table S1). Stained cells were washed with 3 ml 1× PBS, and cells were fixed with eBioscience™ Foxp3 / Transcription Factor Staining Buffer Kit as per manufacturer's guidelines. The fixed samples were passed through a 35-µm filter and resuspended in 300 µl of 1× PBS. 25 µl of counting beads were spiked into each sample before flow cytometry acquisition, to calculate cell numbers per 1 g of tissue. Gating strategy is shown in Fig. S7.

### Cell culture

Primary bone marrow-derived macrophages (BMDMs) were prepared from the femurs and tibias of 3-6-month-old male and female WT and mNLS C57BL/6 mice. Bone marrow was isolated, red blood cells were lysed, and the remaining cells were passed through a cell strainer Cells were cultured in 70% DMEM [containing 10% FBS, penicillin (100 U ml^−1^) and streptomycin (100 µg ml^−1^)] supplemented with L929-conditioned medium for 7 days at 37°C and 5% CO_2_. Cells were seeded at a density of 1×10^6^ cells ml^−1^ for experiments and left to adhere overnight.

Peritoneal macrophages were isolated from the peritoneal lavage. Mice were culled by cervical dislocation, and the peritoneal cavity was lavaged with 5 ml RPMI (containing 3% FBS, 1 mM EDTA). The collected RPMI was centrifuged at 1500 ***g***, 5 min, and the cell pellet resuspended in fresh DMEM [containing 10% FBS, penicillin (100 U ml^−1^) and streptomycin (100 µg ml^−1^)]. Cells were seeded at a density of 1×10^6^ cells ml^−1^ for experiments and left to adhere overnight.

### Cell treatment protocols

For IL-1α priming experiments, WT or mNLS BMDMs or peritoneal macrophages were treated with vehicle (PBS) or LPS (1 µg/ml) for 4 h, or LPS (1 µg/ml) for 0-5 h. Cell lysates and supernatants were subsequently analysed using ELISA, immunofluorescence labelling, qPCR and RNA-Seq.

For IL-1α processing and release experiments, WT or mNLS BMDMs were first primed with LPS (1 µg/ml) or Pam3CSK4 (100 ng/ml) for 4 h. IL-1α processing was then induced by treatment with ionomycin (0.5-10 µM, 1 h), nigericin (10 μM, 1 h), ATP (0.5-5 mM, 1 h), transfection of LPS (2 μg/ml, 18 h) using lipofectamine 3000, or ZVAD (50 μM, 5 h) in the presence or absence of MCC950 (10 µM; MCC), calpeptin (40 µM; Calp), YVAD (100 μM), or necrostatin-1 (50 μM; Nec1). Cell lysates and supernatants were subsequently analysed using ELISA, western blotting, and cell death assays.

### RNA-sequencing analysis

BMDMs were primed with either vehicle (PBS) or LPS (1 µg ml^−1^) for 4 h. Total RNA was extracted using a PureLink RNA mini kit. In the *in vivo* influenza infection model, infected WT, mNLS, and heterozygous control mice were culled on day 7 after infection with the IAV strain X31 (H3N2). One lung lobe was collected and placed in RNA*later* stabilisation solution prior to subsequent RNA extraction. Total RNA was extracted by transferring to a 2 ml tube containing RLT buffer supplemented with β-mercaptoethanol and a single 2 mm stainless steel bead. Tissue was homogenised into suspension using a Qiagen Tissue Lyser. Total RNA was then extracted using the RNeasy Mini Kit according to the manufacturer's protocol. Total RNA was submitted to the Genomic Technologies Core Facility (GTCF) at the University of Manchester. Quality and integrity of the RNA samples were assessed using a 4200 TapeStation (Agilent Technologies) and then libraries generated using the Illumina^®^ Stranded mRNA Prep. Ligation kit (Illumina, Inc.) according to the manufacturer's protocol. Briefly, total RNA (typically 0.025-1 μg) was used as input material from which polyadenylated mRNA was purified using poly-T, oligo-attached, magnetic beads. Next, the mRNA was fragmented under elevated temperature and then reverse transcribed into first strand cDNA using random hexamer primers and in the presence of actinomycin D (thus improving strand specificity whilst mitigating spurious DNA-dependent synthesis). Following removal of the template RNA, second strand cDNA was then synthesised to yield blunt-ended, double-stranded cDNA fragments. Strand specificity was maintained by the incorporation of deoxyuridine triphosphate (dUTP) in place of dTTP to quench the second strand during subsequent amplification. Following a single adenine (A) base addition, adapters with a corresponding, complementary thymine (T) overhang were ligated to the cDNA fragments. Pre-index anchors were then ligated to the ends of the double-stranded cDNA fragments to prepare them for dual indexing. A subsequent PCR amplification step was then used to add the index adapter sequences to create the final cDNA library. The adapter indices enabled the multiplexing of the libraries, which were pooled prior to loading on to the appropriate flow-cell. This was then paired-end sequenced (59+59 cycles, plus indices) on an Illumina NovaSeq6000 instrument. Finally, the output data was demultiplexed and BCL-to-Fastq conversion performed using Illumina's bcl2fastq software, version 2.20.0.422.

### Bioinformatic data analysis and visualisation

All analyses were conducted in R version 2025.05.1+513 (R Core Team, 2025). For principal component analysis (PCA), count matrices were imported into R and annotated with sample metadata (e.g. genotype and treatment groups). Genes lacking annotation were excluded. A variance stabilising transformation (VST) was applied using the DESeq2 package (Love et al., 2014) to normalise raw counts and stabilise variance across expression levels. Principal component analysis was performed with the plotPCA function in DESeq2, and the first two principal components were visualised with ggplot2 (Wickham, 2016), with the percentage of variance explained displayed on each axis. For volcano plots, differential expression results were imported from processed RNA-Seq datasets and pre-formatted to include gene identifiers, log_2_ fold changes, and adjusted *P*-values. Genes with missing information were excluded, and *P*-values equal to zero were replaced by the smallest positive floating-point value in R for numerical stability. Genes were classified as *upregulated* or *downregulated* using thresholds of |log_2_ fold change|≥2 and *FDR*-adjusted *P*-value ≤0.05; all other genes were classified as not significant. Volcano plots were generated with ggplot2 and ggrepel (Slowikowski, 2024), plotting log_2_ fold changes against –log_10_(*P*-adjusted values). Horizontal and vertical dotted lines indicated statistical significance and fold-change cutoffs, respectively. For gene ontology (GO) enrichment, for each dataset, significantly upregulated and downregulated genes (*FDR*≤0.05) were converted from gene symbols to Entrez Gene identifiers using the org.Mm.eg.db annotation package (https://bioconductor.org/packages/org.Mm.eg.db.html; DOI:10.18129/B9.bioc.org.Mm.eg.db). Enrichment analyses were conducted with the compareCluster function from clusterProfiler (Yu et al., 2012), specifying the biological process (BP) ontology. Significant enrichment was defined at *P*≤0.05. Results were visualised as dot plots, highlighting the most enriched terms.

### qPCR

BMDMs from WT and mNLS mice were treated as described above and RNA extracted as done for the RNA-Seq experiment. RNA (300 ng) was converted to cDNA using SuperScript III reverse transcriptase according to manufacturer's instructions. qPCR was performed using Power SYBR Green PCR master mix and 200 nM of each primer using a 7900HT Fast Real-Time PCR System (Applied Biosystems), and 3 μl of 1:10-diluted cDNA was loaded. Samples were run in duplicate or triplicate. Data were normalised to the expression of the housekeeping genes *Hmbs* or *Gapdh*. Expression levels of genes of interest were computed as follows: relative mRNA expression=*E*^−(*C*t of gene of interest)^/ *E*^−(*C*t of housekeeping gene)^, where *C*t is the threshold cycle value and *E* is efficiency. The following primers were used: IL-1α forward, 5′-TCTCAGATTCACAACTGTTCGTG-3′, IL-1α reverse, 5′-AGAAAATGAGGTCGGTCTCACTA-3′; IL-1β forward, 5′-AACCTGCTGGTGTGTGACGTTC-3′, IL-1β reverse, 5′-CAGCACGAGGCTTTTTTGTTGT-3′; Hmbs forward, 5′-GAAATCATTGCTATGTCCACCA-3′, Hmbs reverse, 5′-GCGTTTTCTAGCTCCTTGGTAA-3′; Gapdh forward, 5′-CAGTGCCAGCCTCGTCC-3′, Gapdh reverse 5′-CAATCTCCACTTTGCCACTGC-3′.

To determine lung viral load, RNA was extracted from one lung lobe from WT and mNLS mice infected with IAV, and uninfected heterozygous mice, as described for RNA-Seq. Subsequently, the purified RNA was reverse-transcribed into cDNA using the High-Capacity RNA-to-cDNA™ Kit, following the manufacturer's instructions. Viral cDNA was quantified using a TaqMan real-time PCR assay on a QuantStudio™ 12 K Flex Real-Time PCR System (Applied Biosystems). The assay was designed to quantify the influenza A matrix protein (MP) gene using the following primers and probe: MP forward, 5′-AAGACCAATCCTGTCACCTCTGA-3′, MP reverse, 5′-CAAAGCGTCTACGCTGCAGTCC-3′, MP probe: [6-FAM]-TTTGTGTTCACGCTCACCGTT-[TAMRA]. All samples were analysed in duplicate. Data were normalised to the expression of the housekeeping gene *Hprt1* (4331182, Mm03024075_m1). Expression levels of genes of interest were computed as above.

### Cell death assay

Cell death was determined by measuring lactate dehydrogenase release into the supernatant using a CytoTox 96 Non-Radioactive Cytotoxicity assay (Promega), according to the manufacturer's instructions.

### ELISA

The levels of IL-1α, IL-1β, IL-6 and TNF in the supernatant were measured by ELISA according to the manufacturer's instructions.

### Immunofluorescence assays

Cells were washed once in PBS then fixed in 4% paraformaldehyde (PFA) for 10 min before being incubated in blocking solution (5% BSA, PBS, 0.1% Triton X-100) for 1 h at room temperature. Cells were incubated with goat anti-mouse IL-1α (1:100, 2 μg ml^−1^) or goat anti-mouse IL-1β (1:100, 2 μg ml^−1^) primary antibodies in 5% BSA, PBS, 0.1% Triton X-100 at 4°C overnight. Cells were then washed and incubated with Alexa Fluor™ 594 donkey anti-goat IgG (1:500, 4 µg ml^−1^) secondary antibody for 1 h at room temperature. Nuclei were stained with DAPI (0.5 µg ml^−1^). Coverslips were mounted on slides using ProLong Gold antifade mounting reagent and left to dry overnight.

### Confocal microscopy

Confocal microscopy images were acquired using a 63×/1.40 HCS PL Apo objective on a Leica TCS SP8 AOBS upright confocal microscope with LAS X software (v3.5.1.18803). To prevent interference between channels, lasers were excited sequentially for each channel. The blue diode with 405 nm and the white light laser with the 594 nm laser line was used, with hybrid and photon-multiplying tube detectors with detection mirror settings set appropriately. Z-stacks were acquired with 0.3 µm steps between Z sections. Images were acquired from 3-5 fields of view from each independent experiment.

### Image analysis

Nuclear localisation was quantified manually on FIJI (ImageJ) using maximum projections and expressed as the percentage of total pro-IL-1α or pro-IL-1β fluorescence intensity that co-localised with DAPI signal.

### Western blotting

Supernatant was collected and cells were lysed in lysis buffer (50mM Tris-HCl, 150mM NaCl; Triton-X-100 1% v/v, pH 7.3) containing protease inhibitor cocktail. Lysates were analysed for IL-1α and IL-1β. Equal volumes of lysates or supernatants were loaded. Samples were run on SDS-polyacrylamide gels and transferred at 25 V onto nitrocellulose or PVDF membranes using a Trans-Blot^®^ Turbo Transfer™ System (Bio-Rad). Membranes were blocked in 5% BSA (w/v) or 5% milk (w/v) in PBS, 0.1% Tween (v/v) (PBST) for 1 h at room temperature. Membranes were incubated at 4°C overnight with goat anti-mouse IL-1α (1:1000, 200 ng/ml) or goat anti-mouse IL-1β (1:800, 250 ng/ml) primary antibodies. Membranes were washed in PBST and incubated with rabbit anti-goat IgG (1:1000, 500 ng/ml) in 5% BSA (w/v) in PBST at room temperature for 1 h. Proteins were visualised with Cytiva Amersham ECL Prime Western Blotting Detection Reagent and G:BOX (Syngene) and Genesys software. Membranes were probed with an anti-β-actin antibody (1:20,000) to detect protein levels of the loading control β-actin. Densitometry performed on cell lysates was first normalised to β-actin, and then expressed relative to a control treatment. Densitometry performed on supernatants was not normalised.

### Data analysis

Nuclear localisation quantification from immunofluorescence images is presented as the median±interquartile range (±IQR), with each data point representing a field of view. All other data are presented as the mean±standard error of the mean (±s.e.m.). Data were assessed for normal distribution using Shapiro-Wilk normality test. Parametric data were analysed using two-tailed unpaired *t*-test, or one-way or two-way ANOVA with Dunnett's or Sidak's post hoc test. Non-parametric data were analysed using unpaired two-tailed Mann–Whitney test or Kruskal–Wallis test with Dunn's post hoc test. Western blots are representative of 6-7 independent experiments. Statistical analysis was performed using GraphPad Prism (v10).

## Supplementary Material

10.1242/dmm.052705_sup1 Supplementary information

Dataset 1. RNAseq Flu experiment differential expression raw data

Dataset 2. RNAseq LPS experiment differential expression raw data
