# Supplementary material for: Inflammatory responses following CRISPR modification of the nuclear localisation sequence in endogenous interleukin-1 alpha
Source: Dis Model Mech. 2026 Apr 13;19(4):dmm052705. doi: 10.1242/dmm.052705 (PMC13133774; doi:10.1242/dmm.052705)
Supplement: Supplementary information [file dmm-19-052705-s1.pdf]

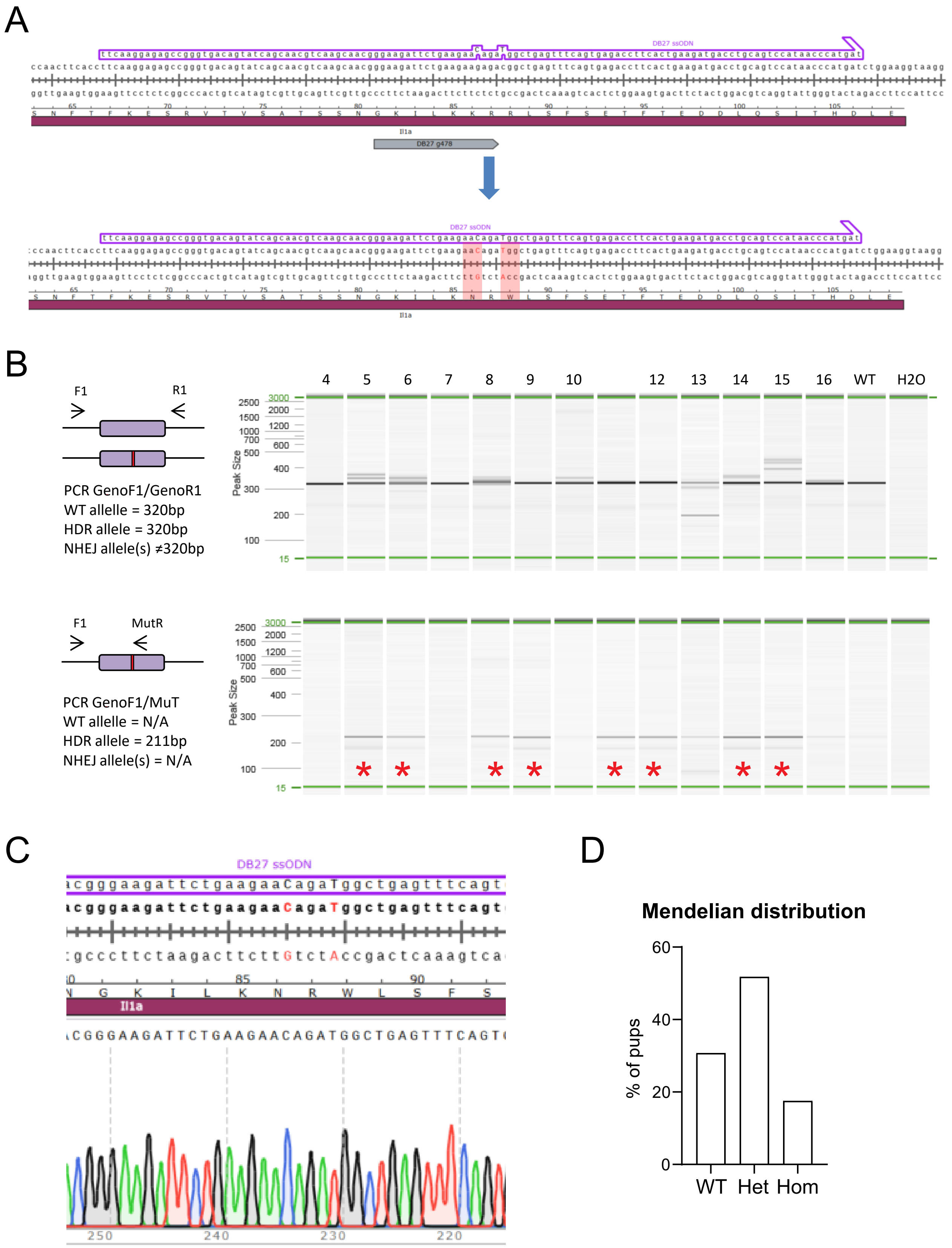

Fig. S1. Design and creation of mNLS mouse. (A) Design of CRISPR modification, indicating sgRNA targeting and ssODN repair template sequence harbouring two base pair changes (upper panel), with predicted sequence following HDR and two amino acid changes in NLS (shaded in red). **(B)** PCR genotyping of F0 pups. PCR Geno F1/GenoR1 (Upper panel) amplifies over the target site (note multiple bands indicates likely NHEJ InDels) and PCR GenoF1/MutR preferentially amplifies candidate HDR alleles, indicated by red asterisks. **(C)** Sanger sequencing of GenoF1/GenoR1 product from Pup 15, indicating correct base pair alterations. **(D)** Genotypes of mice born from all heterozygous-heterozygous breeding pairs during colony breeding (n=35 WT, n=59 Het, n=20 Hom).

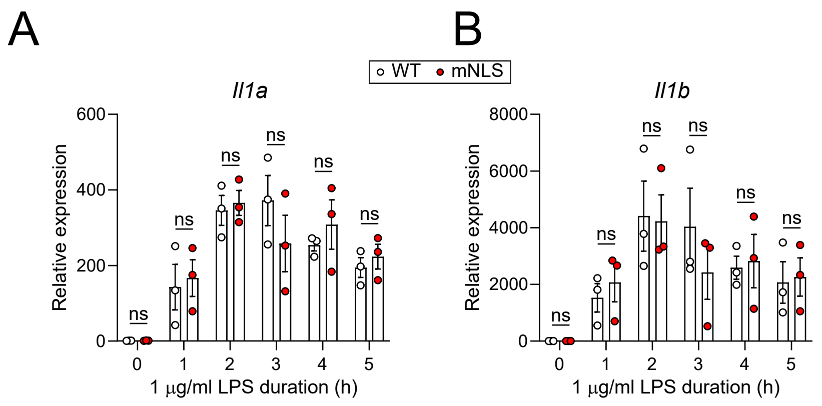

Fig. S2. Pro-IL-1α mNLS mutation does not affect *Il1a* mRNA stability. (A-B) WT or mNLS BMDMs were primed with LPS (1 μg/ml, 0-5 h). qPCR analysis of (A) *Il1a* and (B) *Il1b* expression (n=3). N numbers indicate biological replicates. Data are presented as mean ± SEM. Data were analysed using two-way ANOVA followed by Sidak's post-hoc test. ns, not significant.

Disease Models & Mechanisms • Supplementary information

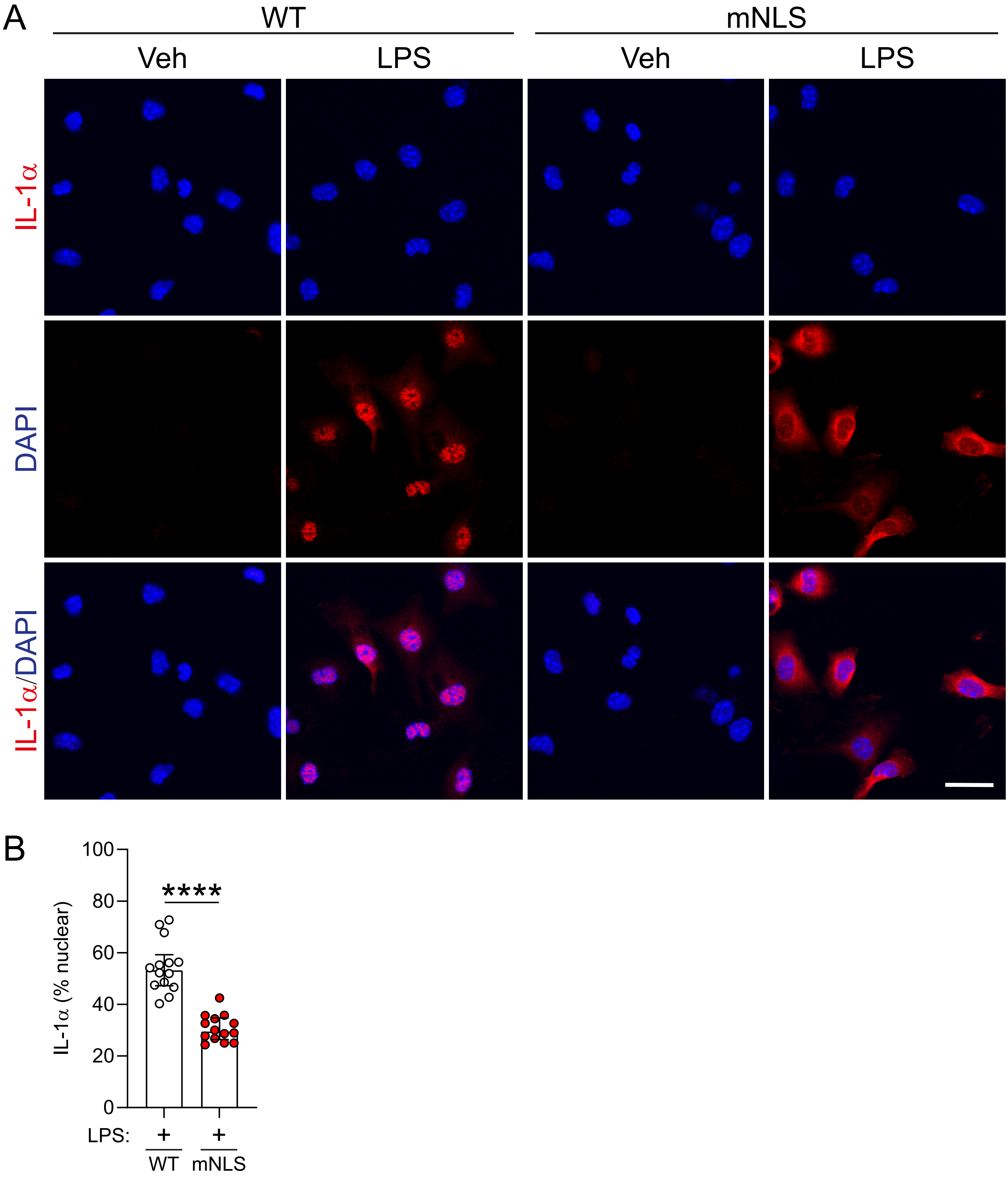

Fig. S3. Pro-IL-1α NLS mutation reduces nuclear localisation in peritoneal macrophages. Peritoneal macrophages were isolated from WT or mNLS mice, and primed with vehicle (PBS) or LPS (1 μg/ml, 4 h). (A) Immunofluorescence labelling of pro-IL-1α, and (B) quantification of nuclear localisation of pro-IL-1α (n=3, with 14 (WT) or 14 (mNLS) fields of view quantified). Scale bar is 20 μm. N numbers indicate biological replicates. Data are presented as median ± IQR. Data were analysed using two-tailed unpaired t-test. ****$P<0.0001$.

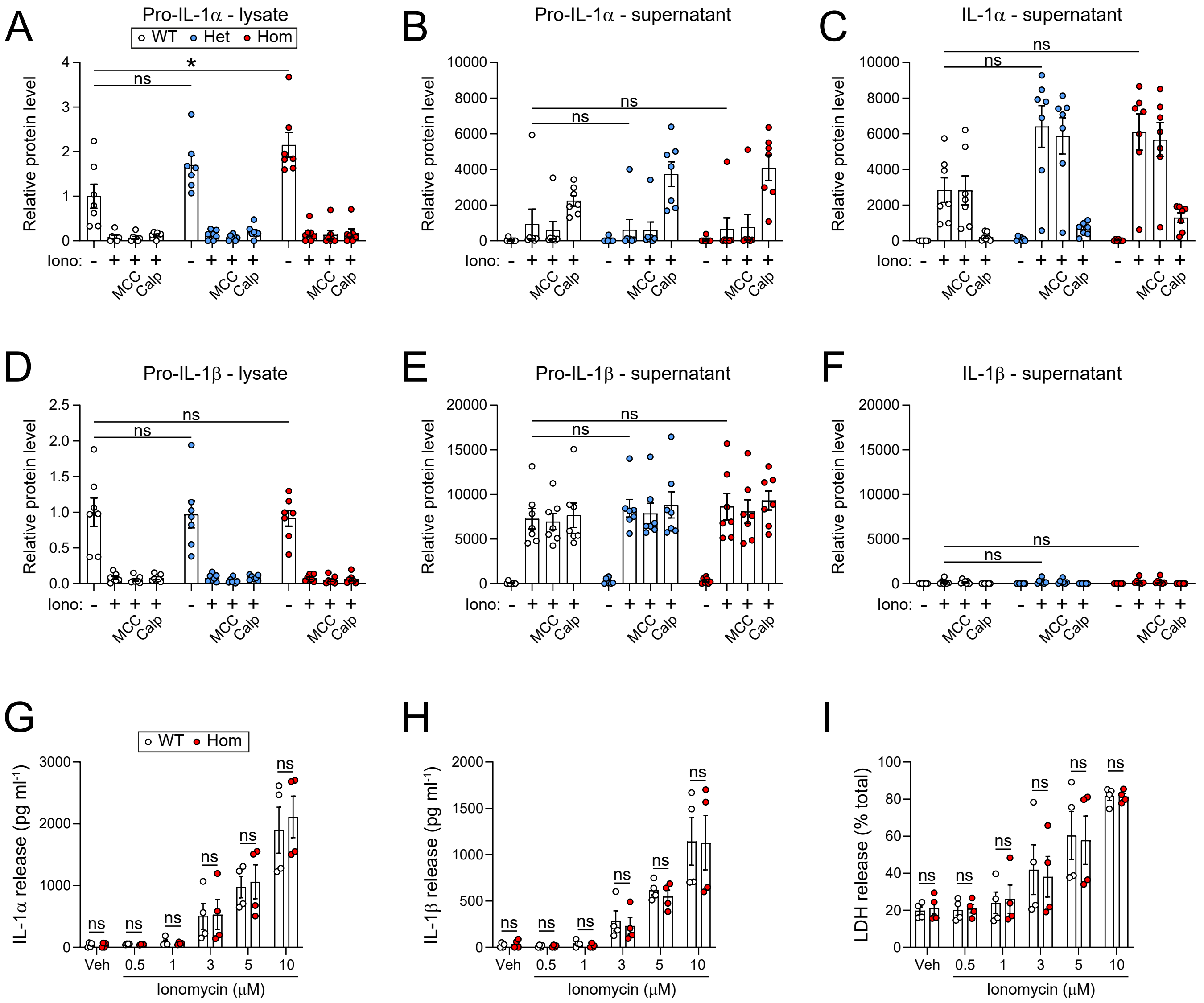

Fig. S4. Pro-IL-1α NLS mutation does not negatively affect processing and release of IL-1α in response to ionomycin. (A-F) Densitometry of western blots from Figure 2D (n=7). (A) Relative pro-IL-1α levels were determined in the lysate, and (B) pro-IL-1α and (C) mature IL-1α levels were determined in the supernatant. (D) Relative pro-IL-1β levels were determined in the lysate, and (E) pro-IL-1β and (F) mature IL-1β levels were determined in the supernatant. **(G-I)** WT or mNLS BMDMs were primed with LPS (1 µg/ml, 4 h), followed by vehicle or ionomycin treatment (0.5-10 µM, 1 h). Supernatants were assessed for (G) IL-1α release, (H) IL-1β release, and (I) LDH release (n=4). N numbers indicate biological replicates. Data are presented as mean ± SEM. Data were analysed using Kruskal-Wallis test followed by Dunn's post-hoc test (A,B,C,E,F,G,H) or one-way ANOVA followed by Dunnett's post-hoc test (D) or Sidak's post-hoc test (I). *P<0.05; ns, not significant.

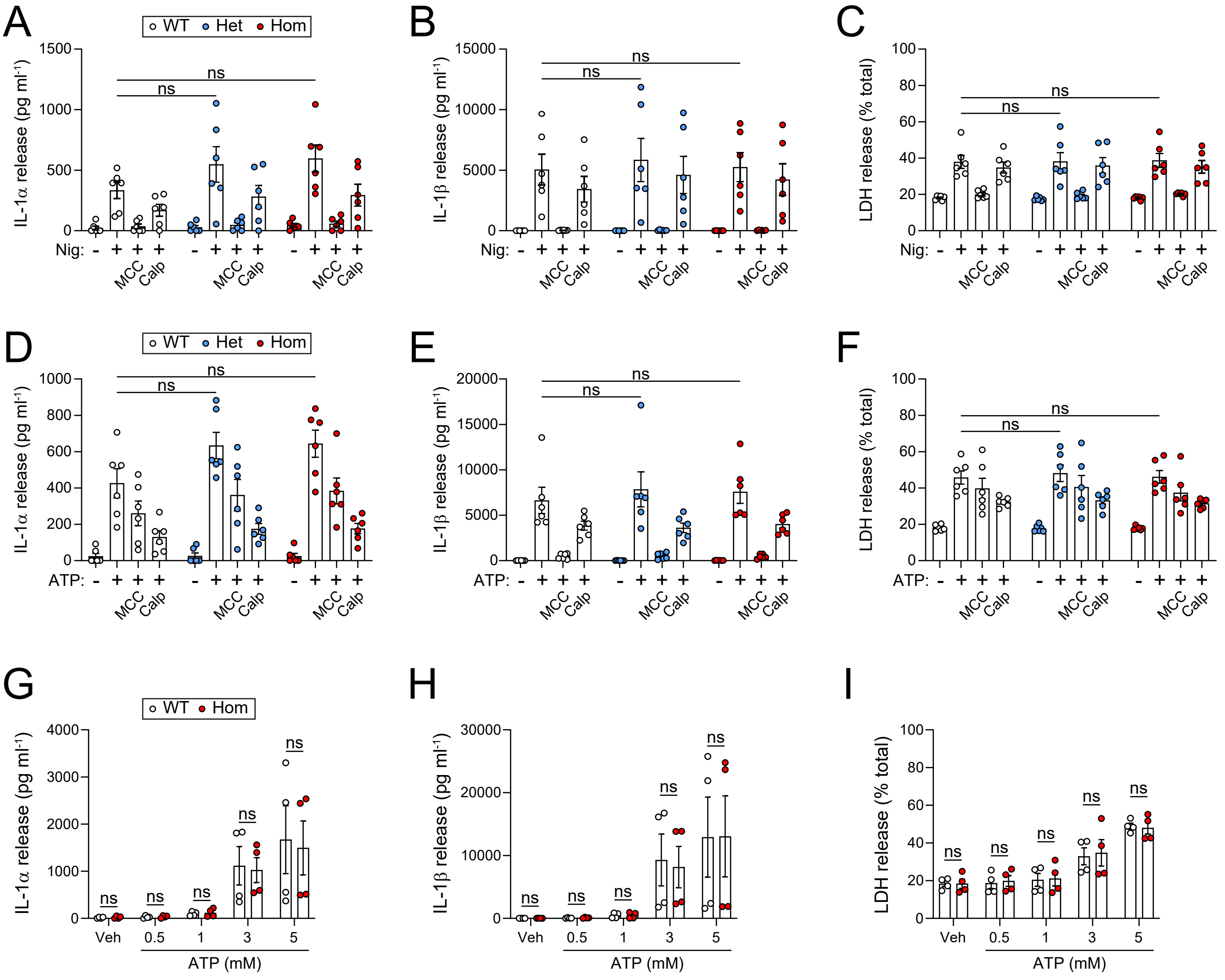

Fig. S5. Pro-IL-1α NLS mutation does not negatively affect processing and release of IL-1α in response to ATP and nigericin. (A-C) WT or mNLS BMDMs were primed with LPS (1 μg/ml, 4 h), followed by nigercin treatment (10 μM, 1 h) in the presence or absence of MCC950 (10 μM; MCC) or calpeptin (40 μM; Calp) (n=6). Supernatants were assessed for (A) IL-1α release, (B) IL-1β release and (C) LDH release. **(D-F)** WT or mNLS BMDMs were primed with LPS (1 μg/ml, 4 h), followed by ATP treatment (5 mM, 1 h) in the presence or absence of MCC950 (10 μM) or calpeptin (40 μM) (n=6). Supernatants were assessed for (D) IL-1α release, (E) IL-1β release and (F) LDH release. **(G-I)** WT or mNLS BMDMs were primed with LPS (1 μg/ml, 4 h), followed by vehicle or ATP treatment (0.5-5 mM, 1 h). Supernatants were assessed for (G) IL-1α release, (H) IL-1β release, and (I) LDH release (n=4). N numbers indicate biological replicates. Data are presented as mean ± SEM. Data were analysed using one-way ANOVA followed by Dunnett's post-hoc test (A,B,D,F) or Sidak's post-hoc test (I), or Kruskal-Wallis test followed by Dunn's post-hoc test (C,E,G,H). ns, not significant.

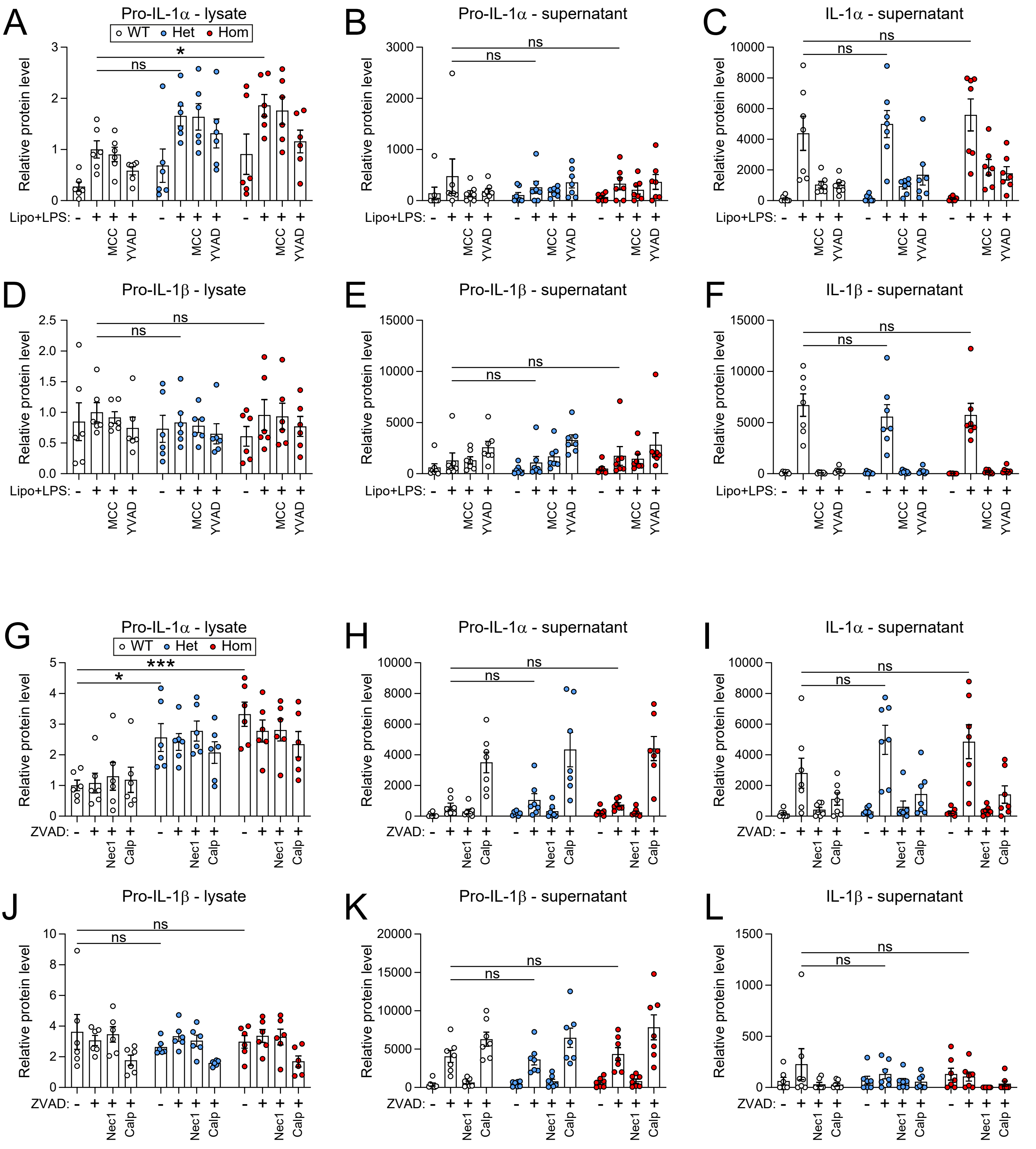

Fig. S6. Pro-IL-1α NLS mutation does not negatively affect processing and release of IL-1α in response to non-canonical inflammasome activation or ZVAD-induced necroptosis. (A-F) Densitometry of western blots from Figure 2H (n=6-7). (A) Relative pro-IL-1α levels were determined in the lysate, and (B) pro-IL-1α and (C) mature IL-1α levels were determined in the supernatant. (D) Relative pro-IL-1β levels were determined in the lysate, and (E) pro-IL-1β and (F) mature IL-1β levels were determined in the supernatant. **(G-L)** Densitometry of western blots from Figure 2L (n=6-7). (G) Relative pro-IL-1α levels were determined in the lysate, and (H) pro-IL-1α and (I) mature IL-1α levels were determined in the supernatant. (J) Relative pro-IL-1β levels were determined in the lysate, and (K) pro-IL-1β and (L) mature IL-1β levels were determined in the supernatant. N numbers indicate biological replicates. Data are presented as mean ± SEM. Data were analysed using one-way ANOVA followed by Dunnett's post-hoc test (A,C,F,G,I,K) or Kruskal-Wallis test followed by Dunn's post-hoc test (B,D,E,H,J,L). ***$P<0.001$; *$P<0.05$; ns, not significant.

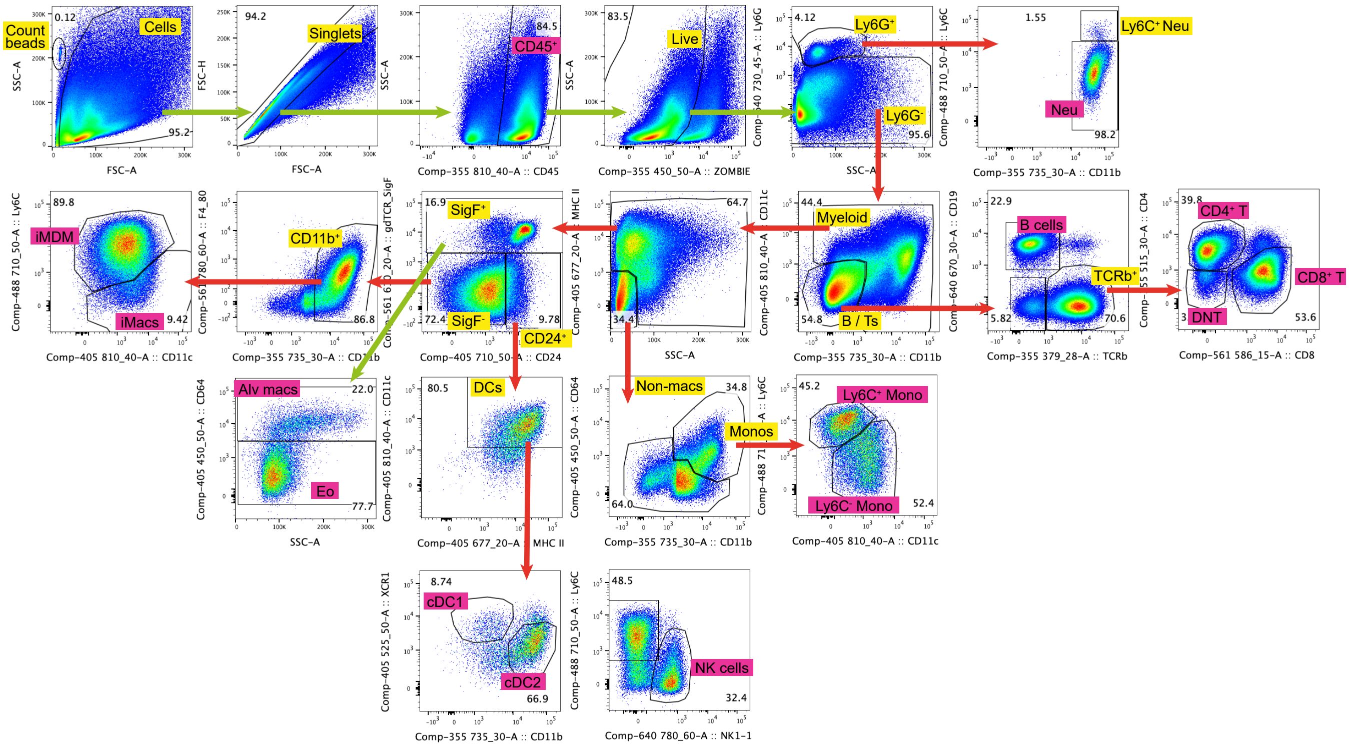

Fig. S7. Flow cytometry gating strategy following in vivo IAV infection. Neu (neutrophils); DNT (CD4⁻ CD8⁻ non-classical T cells); Ly6C⁺ Mono (Ly6C⁺ classical monocytes); Ly6C⁻ Mono (Ly6C⁻ monocytes); NK cells (natural killer cells); cDC1/cDC2 (conventional dendritic cell subtypes 1/2); Eo (eosinophils); Alv macs (alveolar macrophages); iMDM (inflammatory monocyte-derived macrophages); iMacs (interstitial macrophages).

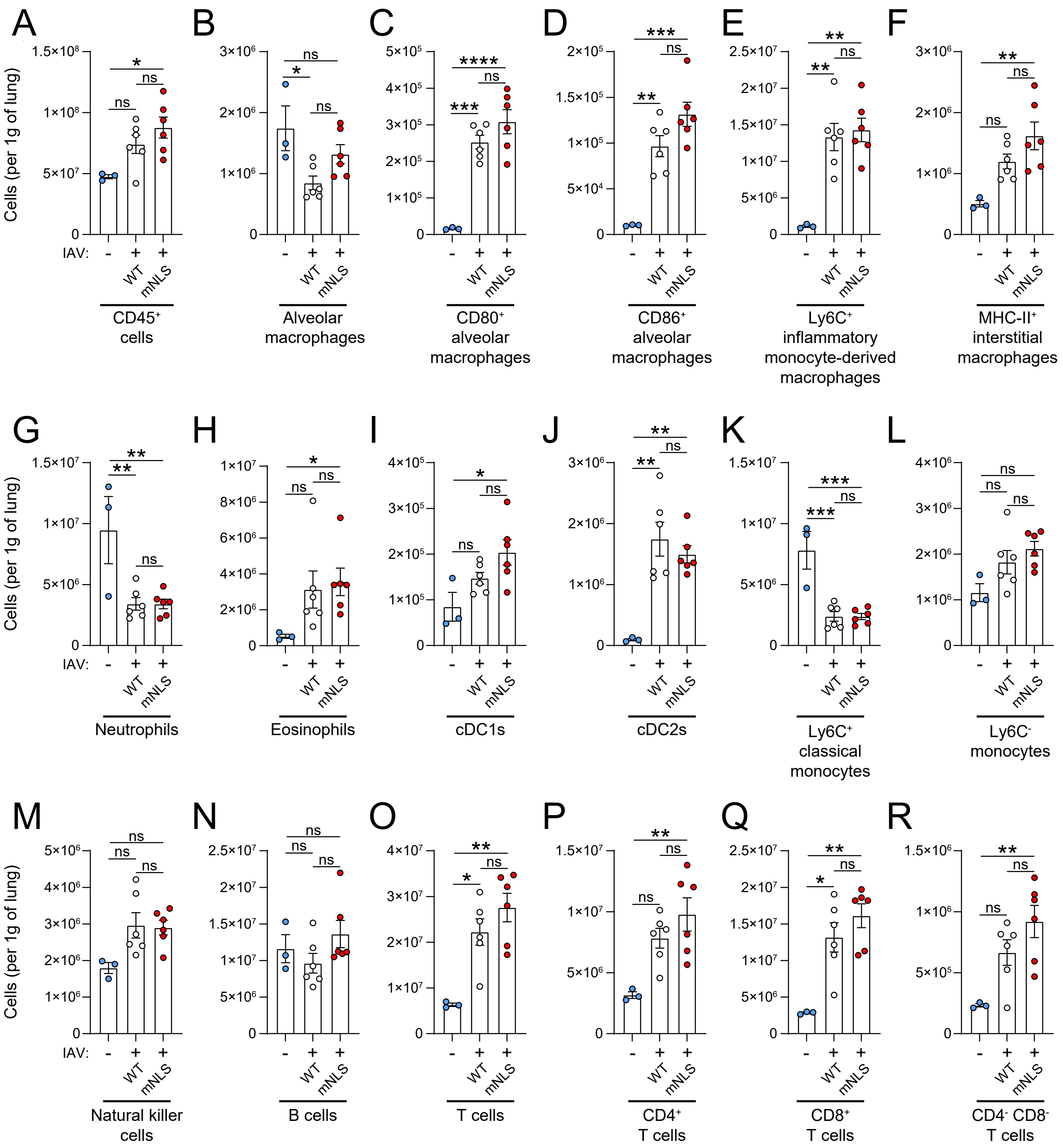

Fig. S8. Immune subset populations following IAV infection in WT and mNLS mice. WT (n=6) or mNLS (n=6) mice were infected intranasally with live influenza strain X31 (10^3 PFU in 30 µl of PBS), while heterozygous mice (n=3) were left uninfected (naïve) as a control. **(A-R)** Cell populations were determined using flow cytometry. See Figure 5. N numbers indicate number of individual mice. Data are presented as mean ± SEM. Data were analysed using one-way ANOVA followed by Sidak's post-hoc test (A-G. I-M, O-R) or Kruskal-Wallis test followed by Dunn's post-hoc test (H,N). ***P<0.0001; ***P<0.001; **P<0.01; *P<0.05; ns, not significant.

Table S1. Flow cytometry antibody panel

Antibody	Clone	Isotype	Dilutions	Conjugate	Company	Catalog #
**Immunophenotyping Panel**						
CD80	16-10A1	Armenian Hamster IgG	1;600	Alexa Fluor® 488	BioLegend	104716
CD86	GL-1	Rat IgG2a, κ	1;600	BV605	BioLegend	105037
Ly-6C	HK1.4	Rat IgG2c, κ	1;600	PerCP/Cy5.5	BioLegend	128012
Ly-6G	1A8	Rat IgG2a, κ	1;600	Alexa Fluor® 700	BioLegend	127622
CD19	6D5	Rat IgG2a, κ	1;600	APC	BioLegend	115512
CD11b	M1/70	Rat IgG2b, κ	1;1000	BUV737	BD Biosciences	612800
CD64 (FcγRI)	W18349C	Rat IgG2a, κ	1;600	BV421	BioLegend	164407
F4/80	BM8	Rat IgG2a, κ	1;600	PE/Cy7	BioLegend	123114
Siglec-F	E50-2440	Rat IgG2a, κ	1;1000	PE-CF594	BD Biosciences	562757
CD11c	N418	Armenian Hamster IgG	1;600	BV785	BioLegend	117336
I-A/I-E	M5/114.15.2	Rat IgG2b, κ	1;1200	BV650	BioLegend	107641
CD24	M1/69	Rat IgG2b, κ	1;800	BV711	BioLegend	101851
XCR1	ZET	Mouse IgG2b, κ	1;400	BV510	BioLegend	148218
CD8a	53-6.7	Rat IgG2a, κ	1;600	PE	BioLegend	100708
**Influenza tetramer and T-cell activation Panel**	**Sequence/Clone**					
Influenza A $NP_{311-325}$	QVYSLIRPNENPAHK	I-Ab	1;200	APC	NIH Tetramer Core Facility, Emory University	
Influenza A $NP_{366-374}$	ASNENMETM	H-2D(b)	1;200	BV421	NIH Tetramer Core Facility, Emory University	
CD11b (Dump)	M1/70	Rat IgG2b, κ	1;600	APC/Cy7	BioLegend	101226
Ly-6G/Ly-6C (Gr-1) (Dump)	RB6-8C5	Rat IgG2b, κ	1;600	APC/Cy7	BioLegend	108424
CD19 (Dump)	6D5	Rat IgG2a, κ	1;600	APC/Cy7	BioLegend	115530
Siglec-F (Dump)	E50-2440	Rat IgG2a, κ	1;600	APC/Cy7	BD Biosciences	565527
CD25	PC61	Rat IgG1, λ	1;600	Alexa Fluor® 700	BioLegend	102024
CD8a	53-6.7	Rat IgG2a, κ	1;600	BV785	BioLegend	100750
CD185 (CXCR5)	2G8	Rat IgG2a, κ	1;400	BV650	BD Biosciences	563981
CD62L	MEL-14	Rat IgG2a, κ	1;600	BUV737	BD Biosciences	612833
CD44	IM7	Rat IgG2b, κ	1;600	PE/Cy7	BioLegend	103030
CX3CR1	SA011F11	Mouse IgG2a, κ	1;600	BV711	BioLegend	149031
PD-1	29F.1A12	Rat IgG2a, κ	1;800	BUV661	BD Biosciences	568603
CD127	A7R34	Rat IgG2a, κ	1;400	PE	BioLegend	135010
KLRG1	2F1/KLRG1	Syrian Hamster IgG	1;400	PerCP/Cy5.5	BioLegend	138418
CD103	2E7	Armenian Hamster IgG	1;400	BV510	BioLegend	121423
CD69	H1.2F3	Armenian Hamster IgG	1;200	BV605	BioLegend	104530
**Common markers**						
CD45	30-F11	Rat IgG2b, κ	1;600	BUV805	BD Biosciences	568336
γδ T-Cell Receptor	GL3	Armenian Hamster IgG2, κ	1;800	PE-CF594	BD Biosciences	563532
TCR β Chain	H57-597	Armenian Hamster IgG2, λ1	1;600	BUV395	BD Biosciences	569248
CD4	GK1.5	Rat IgG2b, κ	1;800	BUV496	BD Biosciences	612952
NK-1.1	PK136	Mouse IgG2a, κ	1;600	APC/Cy7	BioLegend	108724
CD16/CD32 (Fc block)	2.4G2	Rat IgG2b, κ	1;200		BD Biosciences	553142
Live/Dead Fixable Zombie UV			1;1000		BioLegend	423108

Dataset 1. RNAseq Flu experiment differential expression raw data

Available for download at
https://journals.biologists.com/dmm/article-lookup/doi/10.1242/dmm.052705#supplementary-data

Dataset 2. RNAseq LPS experiment differential expression raw data

Available for download at
https://journals.biologists.com/dmm/article-lookup/doi/10.1242/dmm.052705#supplementary-data
